# Epigenetics of Genes Displaying High and Preferential Expression in Myoblasts

**DOI:** 10.3390/epigenomes10010020

**Published:** 2026-03-13

**Authors:** Kenneth C. Ehrlich, Michelle Lacey, Sriharsa Pradhan, Melanie Ehrlich

**Affiliations:** 1 Center for Bioinformatics and Genomics, Tulane University Health Sciences Center, New Orleans, LA 70112, USA; kehrlich@tulane.edu; 2 Department of Mathematics, Tulane University, New Orleans, LA 70118, USA; mlacey1@tulane.edu; 3 Genome Biology Division, New England Biolabs, Ipswich, MA 01938, USA; pradhan@neb.com; 4 Tulane Cancer Center and Hayward Human Genetics Center, Tulane University Health Sciences Center, New Orleans, LA 70112, USA

**Keywords:** whole-genome bisulfite sequencing (WGBS), enzymatic methyl-seq (EM-seq), broad H3K4me3 domains, super-enhancers, enhancers, myoblasts, skeletal muscle, asymmetry enhancer (ASE), cryptic promoters, alternative promoters

## Abstract

Background/Objectives: Genome-wide studies of differential DNA methylation often focus on its role in turning transcription on or off. Here we report some atypical epigenetic/transcription relationships for 92 genes that are highly and preferentially expressed in primary human myoblasts relative to heterologous cell cultures. Methods: We compared methylomes and myoblast-specific differentially methylated regions (DMRs) with methylomes, chromatin profiles, and transcriptomes for many different cell populations. Results: We found that myoblast-associated promoter hypomethylation was unusually prevalent among the 92 myoblast-preferential genes. Sometimes this promoter hypomethylation was seen as a myoblast-associated extension of their constitutively unmethylated region at a CpG island. All 92 genes showed some myoblast-specific hypomethylation, including 32 genes at tissue-specific super-enhancers or broad H3K4-trimethylated promoters. Myoblast hypermethylated DMRs were also associated with almost half of the myoblast-preferential genes. These hypermethylated DMRs were often in intragenic locations embedded in H3K36-trimethylated chromatin in myoblasts. Conclusions: Our analysis suggests that some of the hypermethylated DMRs repress cryptic, alternative, or adjacent promoters. Myoblast hypermethylated DMRs may also downmodulate expression in myoblasts to avoid yet higher RNA levels found in adult or fetal skeletal muscle tissue. The epigenetic insights that were obtained can help elucidate the transcription regulation of some of these genes (e.g., *MUSK*, *RAPSN*, *HEYL*, *SYNPO2*, *SYNPO2L*, *STAC3*, *PITX2*, and *TPPP3*) that are implicated in congenital myasthenic syndromes, myasthenia gravis, muscle repair, heart dysfunction, or cancer. This study supports cell type-specific roles for DNA hypo- and hypermethylation as a modulator of transcription levels, in addition to being an on–off switch during differentiation.

## 1. Introduction

Myoblasts are highly specialized progenitor cells that are needed for formation of prenatal skeletal muscle (SkM) and repair of postnatal SkM [1,2,3,4]. New SkM fibers are generated during embryogenesis by pools of myoblasts fusing with each other to give multinuclear myotubes and then myofibers. Later in prenatal development, many of the myoblasts fuse with the myofibers, which allows myofiber enlargement. Subsequently in the fetus, muscle satellite cells (SkM stem cells) are inserted immediately under the myofiber’s basal lamina [5]. Postnatally, SkM growth results mainly from increases in the size of individual myofibers without the involvement of myoblasts [6]. However, some prepubertal and adolescent SkM growth involves satellite cell activation to form myoblasts that fuse with myofibers. In the adult, satellite cells are activated to form myoblasts during repair of damaged SkM [7]. For these prenatal and postnatal transitions, myoblasts need a specialized gene expression profile that includes genes for cell mobility, cell shape changes, and cell fusion.

We have been studying DNA methylation in myoblasts [8,9,10] because of the especially complex role of myoblasts in development and SkM repair and the importance of DNA methylation in the regulation of gene expression. Mammalian DNA methylation has been increasingly demonstrated to play key roles in differentiation in many cell lineages and at many developmental stages [11,12,13]. Understanding normal development-linked epigenetic changes can aid in designing novel treatments for disease, including for pathologies that are not due to epigenetic abnormalities [14]. Moreover, cancer and many other diseases involve derangements in DNA methylation [11,15,16].

We recently analyzed the methylomes of primary human myoblasts using our myoblast whole-genome bisulfite sequencing (WGBS) and enzymatic methyl-seq (EM-seq) databases [17]. Differentially methylated regions were determined by comparison of myoblast methylomes to those of six diverse types of normal cell cultures to identify myoblast-hypomethylated DMRs (Myob-hypom DMRs) and myoblast-hypermethylated DMRs (Myob-hyperm DMRs). Given the extensive interplay between DNA methylation and chromatin epigenetics [18,19,20], chromatin profiles were compared at the DMRs and in their gene neighborhoods in myoblasts and in many different types of cell cultures and tissues [21,22]. Genome-wide analyses showed that Myob-hypom DMRs were strongly associated with enhancer chromatin (Enh chrom) but only very low percentages were embedded in promoter chromatin (Prom chrom) [17]. Myob-hyperm DMRs were enriched at weak promoter chromatin (weak Prom chrom, H3K4me1 with only a low H3K27ac signal) and repressed chromatin (Repr chrom, H3K27me3 or H3K9me3). We also observed an unexpected enrichment of Myob-hyperm DMRs that overlap sites occupied by CCCTC-binding factor (CTCF), a ubiquitously expressed transcription factor (TF) that is critical for determining the sites of chromatin looping [23].

In the present study, we examined the epigenetics of genes that are expressed highly and preferentially in a very specialized cell type to elucidate the kinds of regulatory strategies that are associated with this class of genes. We focused on 92 genes that were strongly and preferentially expressed in myoblasts compared with many heterologous cell cultures. Among the questions that we addressed about these genes were the frequency of myoblast-associated DNA hypomethylation at promoters vs. enhancers, intragenic vs. intergenic enhancers, standard enhancers vs. super-enhancers (unusually long enhancers [24]), standard promoters vs. broad promoters [25], and promoters with or without CpG islands (CGIs). We also compared DNA hypomethylation and hypermethylation in the vicinity of these genes in terms of frequency, location, and the type of overlapping chromatin. The myoblast methylation/chromatin relationships for this set of genes were compared to the analogous genome-wide data. For 12 of these genes, we studied in detail epigenetic/transcription relationships in myoblasts compared with analogous relationships in many different cell cultures and tissues. The genes examined in detail included ones that pose unusual questions about their epigenetic regulation. Among these genes were a myoblast-preferential coding gene (*B3GALT2*) inside the intron of a broadly expressed gene, genes with different cell/tissue specificity that are unusually close to one another (*SYNPO2L/MYOZ1*; *TPPP3/ZDHHC1*), and a gene that has large regions of hypermethylation including upstream of its promoter that are associated with its expression in myoblasts (*PITX2*). Our findings highlight the associations of high and cell type-specific expression to DNA hypomethylation in broad promoter regions and super-enhancers, as well as the spreading of constitutively unmethylated promoter regions in myoblasts. This study also elucidated various roles that local DNA hypermethylation may play in the control of expression of such highly expressed and cell type-specific genes.

## 2. Results

### 2.1. General Characteristics of Myoblast DMRs Associated with Myoblast-Preferential Genes

We studied DNA methylation associated with coding genes that are highly expressed in myoblasts, with fragments per kilobase million (FPKM) in myoblasts ≥ 20, and that exhibit a strong selectivity for myoblasts, with a ratio of Myob FPKM to the average FPKM for five heterologous cell cultures ≥ 20, according to ENCODE data [22,26]. The heterologous cell cultures were HUVEC, an umbilical vein endothelial primary cell culture; NHEK, an epidermal keratinocyte primary cell culture; NHLF, a lung fibroblast primary cell culture; ESC, an embryonic cell line (H1); and GM12878, a lymphoblastoid cell line (LCL). These were chosen because, as for the primary myoblast cell culture, they are the only cell cultures not derived from cancers that were part of the ENCODE project’s non-strand-specific RNA-seq database [26]. Furthermore, they represent cell types analyzed for transcription by one laboratory and by non-strand-specific RNA-seq, whose quantitation is more accurate than strand-specific RNA-seq.

**Figure 1:**
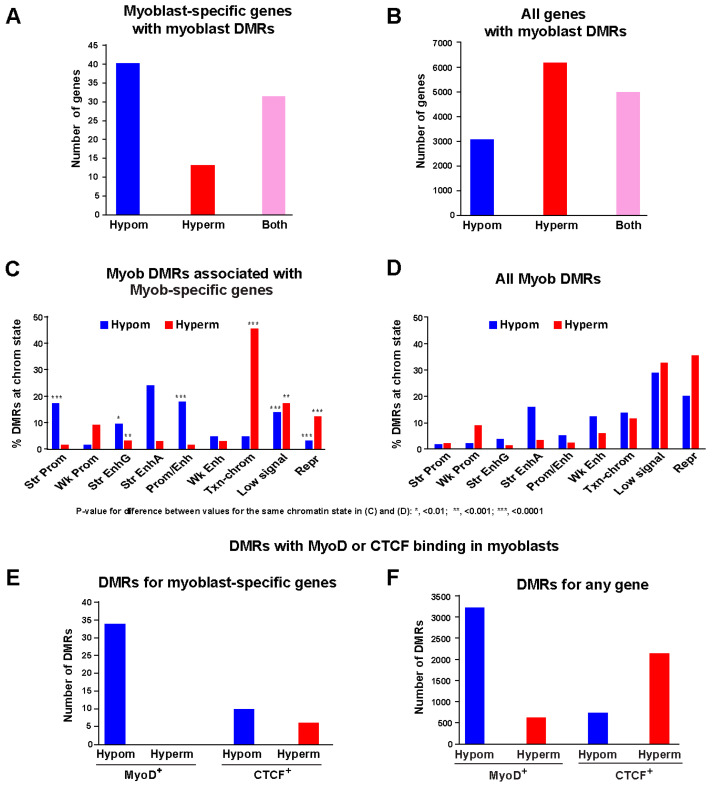
Myoblast preferential DMRs linked to genes highly and preferentially expressed in myoblasts vs. all myoblast DMRs. (**A**) Number of genes with only Myob-hypom DMRs, only Myob-hyperm DMRs, or both among the 85 Myob-preferential genes associated with DMRs. (**B**) Same as for (**A**) but for all Myob DMRs [17]. (**C**) Distribution of Myob-hypom or Myob-hyperm DMRs among different categories of chromatin (18-state model from Roadmap Epigenomics Project). (**D**) Same as for (**C**) but for all Myob DMRs. (**E**) Overlap of Myob-hypom or -hyperm DMRs with MyoD or CTCF binding sites that are occupied in myoblasts. (**F**) As for (**E**) but for all DMRs. We used a threshold methylation difference of |0.25| for these Myob DMRs. Abbreviations: Myob, myoblast; hypom, hypomethylated; hyperm, hypermethylated; Prom, promoter chromatin; Str, strong; Wk, weak; Enh, enhancer chromatin; Txn-chrom, transcription chromatin (enriched in H3K36me3); Repr, repressive chromatin enriched in H3K27me3 or H3K9me3; Low signal, chromatin with low signal for H3K4 methylation, H3K27 acetylation, and for H3K36/H3K27/H3K9 trimethylation.

An advantage of studying methylomes from primary human myoblast cultures is that they, in contrast to SkM tissue, have a very low content of 5-hydroxymethylcytosine (5hmC) [9], thus simplifying methylome analysis. We found 92 such genes (Table S1), which we refer to as Myob-preferential genes. Of these Myob-preferential genes, 85 were associated with myoblast-hypomethylated (Myob-hypom) DMRs and/or myoblast-hypermethylated (Myob-hyperm) DMRs (Figure 1A and Figure S1; Tables S1 and S2). The DMRs were obtained from our reported comparisons of myoblast WGBS and EM-seq profiles with WGBS profiles of non-myoblast cell cultures [17]. The assignment of all DMRs to genes was done by prioritizing their distance to a TSS as described in Methods. We previously showed that EM-seq and WGBS on the same batch of myoblast DNA gave similar results [17]. Thirty-six of the 92 Myob-preferential genes were also preferentially expressed (see Section 4) in SkM. As expected, a gene ontology analysis (Genomic Regions Enrichment of Annotations Tool (GREAT) [27]) showed that terms related to muscle were strongly overrepresented among Myob-DMRs associated with Myob-preferential genes (e.g., 34 genes associated with Muscle Systems Process, binomial *p* = 1 × 10^−34^).

Significant differences in the distributions of various types of chromatin embedded in Myob-hypom or Myob-hyperm DMRs were found when comparing DMRs linked to Myob-preferential genes with all Myob-hypom or Myob-hyperm DMRs (Figure 1C,D, Table S3). Most notably, the Myob-hypom DMRs that overlapped strong promoter-type chromatin (Prom chrom, H3K27ac and H3K4me3 enrichment [21]) were much more prevalent among DMRs linked to Myob-preferential genes than among all Myob-hypom DMRs (Figure 1C,D). This was also the case for Myob-hypom DMRs overlapping Prom/Enh chrom, which is a mixture of strong Prom chrom and strong enhancer-type chromatin (Enh chrom, H3K27ac and H3K4me1 enrichment).

Very strong enrichment of Myob-hyperm DMRs at transcription-type chromatin (Txn-chrom) was seen for Myob-preferential genes vs. all DMRs [17] (Figure 1C,D). Txn-chrom is marked by H3K36 trimethylation, H3K36me3, without H3K27ac enrichment [21]. In addition, there was a small subtype of Enh chrom that is termed EnhG chrom [21], which is enriched for H3K27ac and H3K4me1 in addition to H3K36me3. The other type of Enh chrom is EnhA chrom (H3K4me1 and H3K27ac) which lacks H3K36me3 [21]. For EnhA chrom, there was no significant enrichment in Myob-hypom DMRs linked to Myob-preferential genes vs. in all Myob-hypom DMRs but there was for EnhG chrom. Myob DMRs linked to Myob-preferential genes were depleted in both Repr chromatin (enriched in H3K27me3 or H3K9me3) and Low signal chromatin (little or no H3K4me3, H3K4me1, H3K9me3, H3K36me3, or H3K27ac) relative to all DMRs.

The distribution of Myob-hypom DMRs relative to the TSS was different from that of Myob-hyperm DMRs, as expected (Figure S2). In addition, 31% (29) of the Myob-hypom DMRs were present 2 kb upstream to 2 kb downstream of the transcription start site (TSS − 2kb to +2 kb) of Myob-preferential genes compared with only 5% of the total 19,635 Myob-hypom DMRs (Figure S2). Binding of MyoD, the critical SkM-lineage-specific transcription factor (TF) that acts in a mostly positive manner [28,29], was prevalent at Myob-hypom DMRs but was not seen at any hyperm DMRs linked to Myob-preferential genes (Figure 1E,F). Similarly, in our previous study of all Myob DMRs, motifs recognized by MYOD were the most highly overrepresented TF motifs among Enh chrom-overlapping Myob-hypom (but not Myob-hyperm) DMRs [17]. The architectural TF, CTCF, bound within many Myob-hypom and hyperm DMRs (Figure 1E,F). CTCF can bind to methylated regions and even methylated recognition sites depending on exactly where the methylation is located [17,30,31]. The number of Myob-hypom DMRs that were MyoD^+^ in myoblasts greatly exceeded those that were CTCF^+^ for both Myob DMRs associated with Myob-preferential genes and for all Myob DMRs. The depletion of CTCF^+^ hyperm DMRs associated with Myob-preferential genes vs. all Myob-hyperm DMRs (Figure 1E,F) might be due to a decreased need for restraining formation of Enh chrom or Prom chrom adjacent to CTCF sites at very highly expressed genes [17].

### 2.2. Hypomethylation at Non-CpG Island Promoters

**Figure 2:**
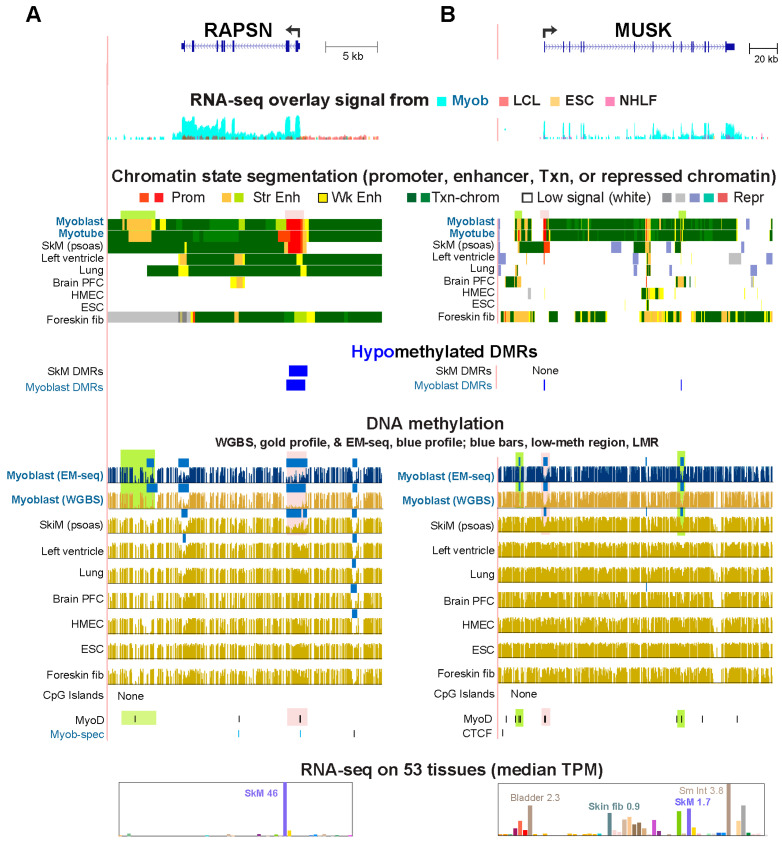
Promoter hypomethylation in myoblasts is associated with high and preferential expression of *RAPSN* and *MUSK* in myoblasts. Both genes encode proteins that interact with each other and are required for clustering of nicotinic acetylcholine receptors at the neuromuscular junction. (**A**) *RAPSN* at chr11:47,452,142–47,478,510. (**B**) *MUSK* at chr9:113,397,994–113,596,335. Transcription start sites and direction are indicated by broken arrows. Pink and green highlighting, locations of Myob-hypom DMRs or LMRs (low-methylated regions vs. the same genome) cited in the text. The cell culture RNA-seq track shows overlapping, color-coded, log-scale signal profiles. Myob, myoblasts; LCL, a lymphoblastoid cell line; ESC, embryonic stem cell line; NHLF, lung fibroblast cell culture; SkM, skeletal muscle; HMEC, mammary epithelial cell culture; fib, fibroblasts; PFC, prefrontal cortex; Sm Int, small intestine; low-meth, low-methylation. CTCF/Myob-spec track: blue bars, CTCF sites seen in myoblasts but not in HMEC or ESC. Coordinates are for hg19; tracks are from the UCSC Genome Browser including for GTEx RNA-seq on tissues (linear scale) for this and subsequent gene figures.

Almost all of the 40% (38) of the Myob-preferential genes that had a CGI promoter were constitutively unmethylated, as expected. Only 16 of 259 Myob DMRs had at least 30% overlap with a CGI (Table S2). One of these was a Myob-hypom DMR, and the rest were Myob-hyperm DMRs. Twenty-nine Myob-preferential genes, including *RAPSN* and *MUSK*, had a Myob-hypom DMR at a non-CGI promoter region (TSS −2 kb to +2 kb region), and 14 of these displayed a MyoD^+^ Myob-hypom DMR at their promoter region, like *RAPSN* and *MUSK* (Figure 2, pink highlighting, and Table S2). Both of these genes were preferentially expressed in SkM as well as in myoblasts (Figures S3A and S4; Table S1).

*RAPSN* encodes a postsynaptic scaffold protein with E3 ligase function (RAPSN/RAPSYN) that is needed at the neuromuscular junction but also plays a role in subcellular organization in myoblasts [32,33]. Normal functioning of RAPSN protein at the neuromuscular junction requires MUSK, a receptor tyrosine kinase [34]. Both the 5′ hypomethylation and the Prom chrom were mostly downstream of the TSS (Figure 2, pink highlighting). These genes also displayed Myob-associated hypomethylation that was seen as low-methylation regions (LMRs, Figure 2, blue bars in DNA methylation tracks) relative to the same genome [35], which were observed preferentially in myoblasts and SkM. Several of these LMRs were at Enh chrom in myoblasts were MyoD^+^ (Figure 2, green highlighting).

The steady-state levels of RNA in myoblasts from the small *RAPSN* gene (11 kb) were about five-fold higher than for the larger *MUSK* gene (138 kb), which might be explained by differences in MyoD and CTCF binding at their promoters. The MyoD signal for *RAPSN* promoter binding in myoblasts was about 6-fold stronger than for the *MUSK* promoter although both genes had MyoD binding to consensus MyoD sites within 0.1 kb upstream of their TSS. Only the *RAPSN* promoter contained a CTCF binding site occupied in myoblasts (Figure 2), and this site was specific for myoblasts relative to all other normal cell populations in the Unibind database [36]. Furthermore, the MyoD site at the *RAPSN* promoter overlaps the 3′ base of the CTCF consensus sequence. Interaction between MyoD and CTCF was found to be important in establishing some of the chromatin loops between promoters and enhancers in the SkM lineage [37].

Enh chrom within the *MUSK* gene body and upstream of the promoter was seen in foreskin fibroblasts, which do not express *MUSK* (Figure 2B and Figure S5). This Enh chrom correlates with the preferential expression of *SVEP1*, the *MUSK*-upstream gene, in foreskin fibroblasts. In these cells the *MUSK* promoter region overlaps only Low signal chrom (Figure 2B). This quiescent chromatin and the high methylation at the *MUSK* promoter in foreskin fibroblasts may facilitate the use of part of the large *MUSK* gene body for *SVEP1* enhancers without activating appreciable *MUSK* transcription.

### 2.3. Hypomethylation at Super-Enhancers, Standard Enhancers, Broad Promoters and Standard Promoters

**Figure 3:**
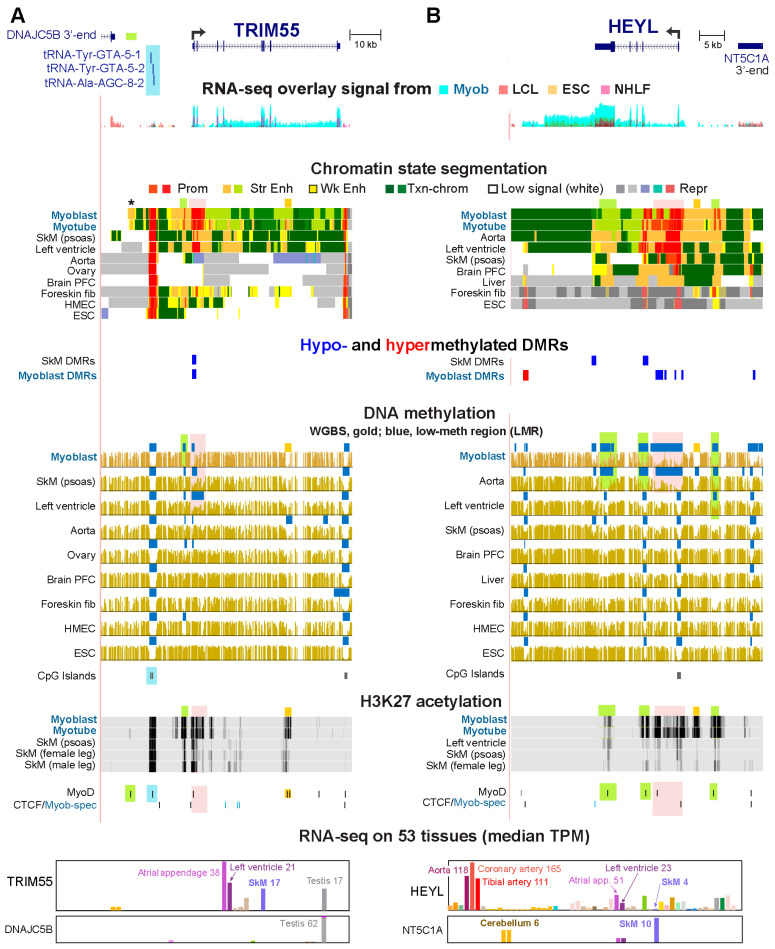
Hypomethylation at *TRIM55* and *HEYL* in myoblasts and heart, aorta, or skeletal muscle. (**A**) *TRIM55* at chr8:67,008,814–67,091,581. Blue highlighting, position of a cluster of tRNA genes; *, position of Enh chrom that is specific for myoblasts/myotubes compared with 100 other Roadmap Project samples. (**B**) *HEYL* at chr1:40,072,857–40,121,594. Pink and green highlighting in both panels, the location of promoter and enhancer chromatin DNA hypomethylation in myoblasts and tissues that highly express these genes; orange boxes above the myoblast WGBS track, additional small regions of myoblast-associated DNA hypomethylation that did not meet the definition of a myoblast DMR or LMR but that are clearly visible in comparisons of WGBS profiles. Labels are like those in the legend for Figure 2. The vertical viewing range for H3K27ac tracks was 0–10.

Eighteen Myob-preferential genes displayed DNA hypom at Myob super-enhancers and 21 genes at standard Enh chrom in myoblasts (Table S4). *TRIM55* and *HEYL* displayed extensive Enh chrom in myoblasts (Figure 3). A small portion of their Enh chrom overlapped regions of myoblast DNA hypomethylation, and this hypomethylation was both intragenic and intergenic, as seen among the Myob-preferential genes in general (Figure 3 and Figure S2). However, most cell/tissue-specific H3K27ac peaks at these two genes overlapped myoblast-associated DNA hypomethylation (Figure 3, highlighting). The large extent of clustering of H3K27ac-enriched chromatin for *HEYL* (although not for *TRIM55*) indicated that *HEYL* overlapped a super-enhancer [24].

*TRIM55* encodes an E3 ubiquitin ligase that is implicated in regulating SkM and cardiac muscle expression as well as in the differentiation of myoblasts to myotubes [38,39]. *HEYL* codes for a TF that is a downstream effector of Notch signaling and can heterodimerize with HES1 to repress *MYOD1* transcription in murine muscle stem cells (satellite cells) [40]. However, *HES1* RNA, unlike *HEYL* RNA, was present at only low levels in human primary myoblasts (FPKM, 2.6 and 122, respectively, Table S1), making a repressive function for HEYL in human myoblasts unlikely.

DNA hypomethylation at Prom and Enh chrom associated with *TRIM55* and *HEYL* was seen specifically in cell populations that highly expressed these genes (Figure 3A and Figure S4). *HEYL*’s promoter overlaps a 0.7 kb CGI that is constitutively unmethylated. On either side of the CGI were small Myob-hypom DMRs at CGI shores (regions within 2 kb of either end of a CGI). Fifteen other Myob-preferential genes had Myob-hypom DMRs overlapping a CGI shore (Table S2). An additional indication of transcription-related spreading of DNA hypomethylation from the *HEYL* 5′ CGI island was seen in myoblasts as well as aorta, which also highly expresses this gene. In their *HEYL* promoter region, the LMR had widened to 8 kb and overlaid mostly Prom chrom. Myob-associated extensions of a constitutive LMR at the promoter region were observed in 18 other Myob-preferential genes (Tables S1 and S4). Moreover, this broad H3K4me3 domain was embedded in the larger myoblast and aorta super-enhancer. In addition to the myoblast hypomethylation associated with *HEYL*, there was a single Myob-hyperm DMR, which might regulate a cryptic intergenic promoter because it overlaps bivalent promoter chromatin in foreskin fibroblasts and ESC.

A surprising feature of *TRIM55* was Myob/myotube-specific Enh chrom 19 kb upstream of the *TRIM55* TSS (Figure 3A, asterisk) despite the presence of a cluster of three broadly expressed tRNA genes between the Enh chrom and the *TRIM55* TSS (Figure 3A, blue highlighting). This Enh chrom did not overlap a Myob-hypom DMR or LMR but there was a myoblast/myotube-specific DNaseI hypersensitive site (Figure S6) and a myoblast-occupied MyoD^+^ site there (Figure 3A, green highlighting). The cell-type-specificity of the Myob/myotube Enh chrom upstream of *TRIM55* did not correlate with the expression profile of the next *TRIM55*-upstream gene, *DNAJC5B*. This suggests that the far-upstream Enh chrom may help modulate expression of *TRIM55* specifically in myoblasts despite the intervening constitutive CTCF site and tRNA gene cluster.

### 2.4. Alternative Promoter Usage and DNA Hypomethylation

**Figure 4:**
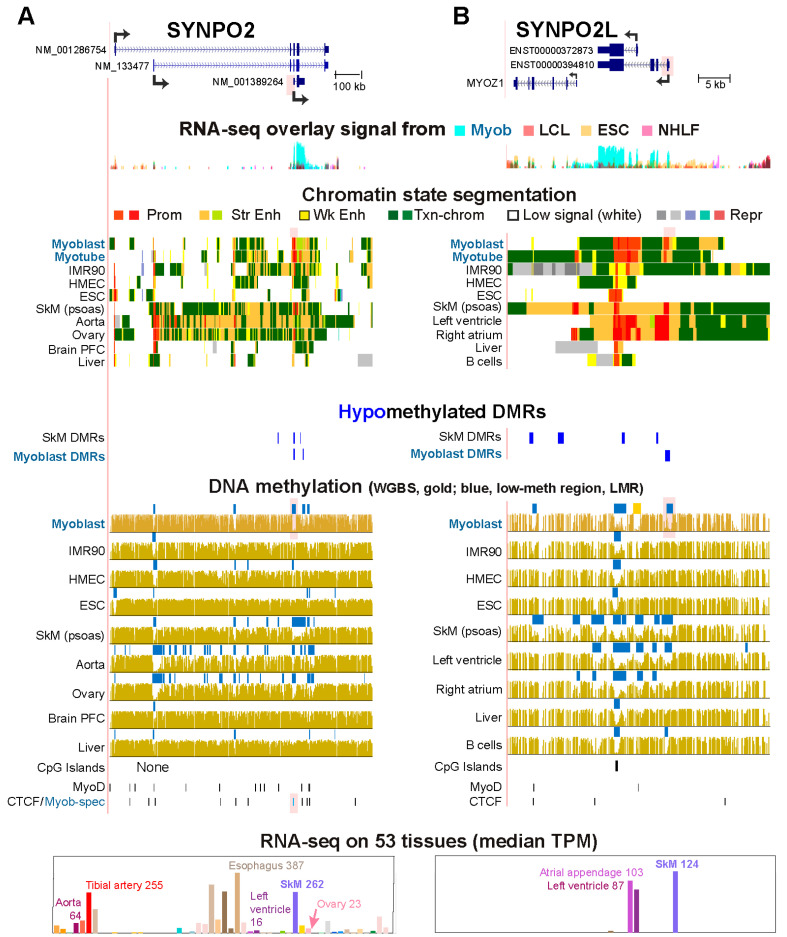
DNA hypomethylation associated with alternative promoter usage for paralogs *SYNPO2* and *SYNPO2L*. (**A**) *SYNPO2* at chr4:119,766,894–120,025,504. NM_00133477 is the RefSeq Select isoform. (**B**) *SYNPO2L* at chr10:75,390,271–75,431,703); Ensembl 115 Basic Isoforms are shown because they matched the RNA-seq data better than RefSeq Curated isoforms. Pink highlighting, the position of the Myob-hypom DMRs that overlap an alternative promoter; orange box in the WGBS tracks in panel (**B**), a region of low methylation in myoblasts at one of the two alternative promoters and overlapping Prom/Enh chrom in myoblasts that did not meet the criteria to be designated a DMR or LMR. Labels are like those in the legend for Figure 2.

Alternative promoter usage was associated with a Myob-hypom DMR for three genes, *SYNPO2*, *SYNPO2L*, and *ARPP21* (Figure 4 and Figure S7; Tables S1 and S2). We examined *SYNPO2* and *SYNPO2L* in detail. These paralogs encode members of the podin family of proline-rich actin-binding proteins. Their protein products, Synaptopodin 2 and Synaptopodin 2 Like, are involved in actin binding and Rho protein signal transduction [39] in both heart and SkM, and for *SYNPO2*, also in smooth muscle [41]. Mutation of *SYNPO2L* is implicated as a risk factor for atrial fibrillation [42]. The *SYNPO2* promoter that is used predominantly in myoblasts is the proximal promoter, which exhibited a Myob-associated CTCF site in a Myob-hypom DMR (Figure 4A, pink highlighting). In contrast, in aorta and ovary, the central promoter had most of the *SYNPO2* Prom chrom and mapped transcription start sites (5′ cap analysis of gene expression (CAGE) [43]. The distal promoter was the predominant one used in ESC (Figure 4A). Tissue-specific alternative promoter usage was reflected in the extents of DNA hypomethylation at the three *SYNPO2* promoter regions. SkM had a wider region of promoter hypomethylation than did myoblasts although both had high levels of expression of the gene and similar amounts of promoter chromatin at their main, proximal promoter. This might reflect a general trend toward increased spreading of DNA hypomethylation in SkM tissue relative to that in myoblasts [8].

*SYNPO2L* has two alternative promoters, both of which are extensively used in myoblasts while the distal promoter is used predominantly in SkM (Figure 4B and Figure S7). The relative levels of usage of the two promoters varied among different heart chambers. In the left ventricle, the distal promoter was favored to encode the longer protein isoform, while in the right atrium, the proximal promoter, which specifies the shorter protein isoform, was favored (Figure S6). Accordingly, left ventricle had less DNA methylation at the distal promoter than did the right atrium (Figure 4B). Myoblasts exhibited hypomethylation at both promoters, although only the distal promoter’s hypomethylation was classified as a Myob DMR (Figure 4B, pink highlighting and orange square). At the distal promoter, methylation was generally inversely related to promoter usage, while at the proximal promoter it was inversely related to either promoter usage or the presence of a peak of H3K27ac in Enh chrom. The high levels of expression in SkM and heart are reflected in their super-enhancers and tissue-specific LMRs.

*MYOZ1* is the nearby, tail-to-head neighbor of *SYNPO2L* (Figure 4B) and encodes an important structural protein for SkM and heart [44]. Only SkM expresses *MYOZ1* at very high levels (TPM, transcripts per kilobase million, 2303; Figure S7). DNA hypomethylation (DMRs or LMRs) at the 5′ end of *MYOZ1* and at an Enh chrom subregion within the gene was seen in SkM. Right atrium, but not left ventricle, displayed an LMR at the *MYOZ* promoter. This correlates with the finding that the right atrial appendage, unlike left ventricle, has considerable expression of *MYOZ* (transcripts per million, TPM, 35 and 1, respectively). The clustering of Enh chrom and DNA hypomethylation in the *MYOZ1/SYNPO2L* neighborhood in SkM suggest that the SkM super-enhancer spanning these genes promotes the expression of both of them even though *MYOZ1* is expressed at a 19-fold higher level in SkM relative to *SYNPO2L* (Figure S7).

### 2.5. Genes That Are Highly and Preferentially Expressed in Myoblasts and Lacked Myoblast DMRs but Displayed Myoblast-Preferential LMRs

**Figure 5:**
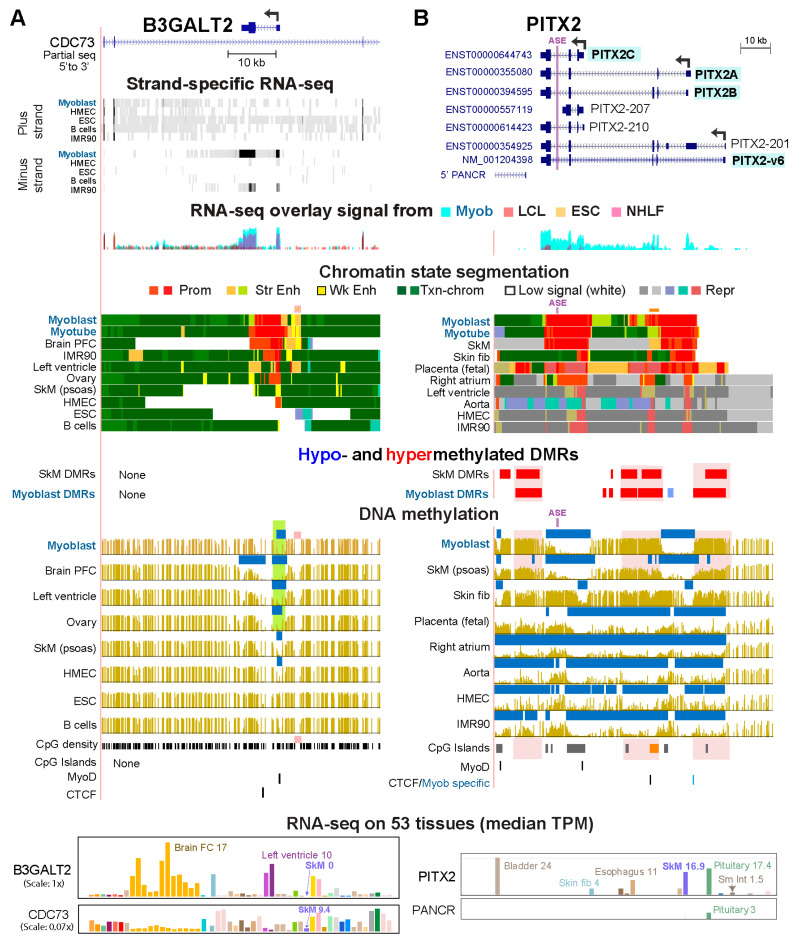
Unusual examples of myoblast differential methylation at Myob-preferential genes: *B3GALT2*, a gene within a gene, and *PITX2*, a gene with predominant myoblast hypermethylation. (**A**) *B3GALT2* at chr1:193,118,998–193,176,515. *B3GALT2* is antisense to its host gene, *CDC73*. Green highlighting, expression-associated hypomethylation at the *B3GALT2* promoter; orange square in CpG density track, a subregion lacking CpGs and overlapping Myob Enh chrom. (**B**) *PITX2* at chr4:111,532,405–111,569,454. Skin fib, postnatal skin fibroblasts used for WGBS, GEO SRX23363337; RNA-seq on 53 tissues (GTEx) includes skin fibroblasts as shown. Pink highlighting, domains of Myob-hypermethylation at *PITX2*; purple line, the *PITX2* ASE (asymmetry enhancer); light blue box in myoblast DMR track, a hypometh DMR with a methylation difference of only −0.26, unlike the threshold of |0.35| for the other DMRs in the figures; orange CpG island, a CGI discussed in the text and whose position is indicated by a horizontal orange line above the myoblast chromatin track. Labels are like those in the legend of Figure 2.

Twenty of the 92 Myob-preferential genes did not exhibit Myob-hypom DMRs. Instead, they displayed myoblast-preferential hypomethylation that did not fit the criteria for a Myob-hypom DMR (Tables S1 and S5). Eight of these genes exhibited myoblast-associated extensions of a constitutive LMR at a promoter, as seen for *FNDC5* and *ADAMTS5* (Figures S8 and S9). Three of these genes and 12 other genes, including *B3GALT2*, displayed a myoblast-associated LMR which was highly methylated in most other cell populations (Table S5). *B3GALT2* has the interesting characteristic of being a cell type-associated gene residing inside a large antisense intron of a constitutively expressed gene, *CDC73* (Figure 5A).

### 2.6. Alternative Promoter Usage and DNA Hypermethylation

Among the Myob-preferential genes linked to Myob-hyperm DMRs were four (*PITX2*, *DOK7*, *IGF2*, and *PTGDS*) whose hypermethylation was associated with tissue-specific use of documented alternative promoters. We examined *PITX2* in detail because of its extensive myoblast hypermethylation and its protein’s function as a homeobox TF with especially diverse roles in development and disease [45]. PITX2 acts as an activator or repressor TF. It is essential for normal SkM formation, regulation of SkM homeostasis and regeneration, and proliferation or differentiation in certain cell lineages, including myoblasts [45,46]. *PITX2*’s tissue specificity includes specific expression in the left atrium [47], except in response to injury [45]. Normal tissues from human left ventricle, right ventricle and right atrium show negligible postnatal expression of *PITX2* (Table S1). However, it is expressed at approximately 100 times higher levels in left atrium (for which epigenomic profiles were not available) than in the other heart chambers, although lower than in SkM [47,48].

*PITX2* has four main transcripts, *PITX2A*, *PITX2B*, *PITX2C*, and *PITX2-v6* RNAs (Figure 5B) generated by differences in alternative promoter usage or RNA splicing and encoding several protein isoforms [49]. In human myoblasts, the *PITX2C* and *PITX2A/PITX2B* promoters were used and displayed broad Prom chrom domains that were bordered by hyperm DMRs (Figure 5B). In the *PITX2C* promoter region, there is the important asymmetry enhancer (ASE; Figure 5B, purple line) that specifies leftward asymmetric orientation of several body cavity organs during organogenesis [50]. The ASE is unmethylated in myoblasts and SkM but methylated in most other cell populations (Figure 5B). In myoblasts, the ASE is probably non-functional as a determinant of asymmetry. This would be consistent with our finding that myoblasts and SkM lack *FOXH1* RNA, which encodes an essential TF for ASE function [50].

Compared with myoblasts, postnatal skin fibroblast primary cultures display lower *PITX2C* expression (Figure 5B, bottom) and had smaller promoter-region LMRs and Prom chrom segments (Figure 5B). In contrast to the other samples, fetal placenta (16 week), which strongly expresses *PITX2* (Figures S4 and S10), used the far-distal, *PITX2-v6* promoter as well as the other two promoters and lacked methylation at all three promoters. Fetal placenta, like non-expressing cell populations, had low or scattered methylation throughout the whole *PITX2* region (Figure 5B and Figure S8). However, for *PITX2* in placenta, the low-methylation DNA domain was associated with a super-enhancer rather than with repressed chromatin. The main protein product from RNA transcribed from the *PITX2-v6* promoter is predicted to be the same as that encoded by the *PITX2B* transcript. Nonetheless, changes in the 5′ untranslated region of a mRNA can be consequential to mRNA stability, pre-mRNA splicing, and internal ribosome entry sites [51].

Postnatal SkM displayed more *PITX2C* than *PITX2A/PITX2B* RNA, which could be related to its Repr chrom downstream of *PITX2A* promoter (Figure 5B, Figures S10 and S11). Fetal SkM, like myoblasts, generated similar amounts of *PITX2C* and *PITX2A/PITX2B* RNA (Figure S10). The hyperm DMR bordering the downstream end of the *PITX2A/PITX2B* promoter in Myob and postnatal SkM overlapped a CGI and weak or strong Prom or Enh chrom in myoblasts and SkM (Figure 5B, orange box and line). This hyperm DMR may be needed to silence the transcription-initiation ability of the CGI in these chromatin contexts. The Myob/SkM hyperm DMR at the 3′ end of *PITX2* covers the TSS for isoforms of the non-coding gene *PANCR* (Figure S11) and might be necessary for silencing transcription from this region when it is in a cell population with very active, adjacent promoters. Of the four tissues in the GTEx database that express *PITX2* at the highest levels (bladder, pituitary, SkM, and esophagus), only pituitary showed appreciable RNA signal for *PANCR* (Figure 5B). CAGE analysis indicated that the 5′ end of *PANCR* isoforms was used for transcription initiation in pituitary, left atrium, and placenta but not in myoblasts (Figure S11, top row of CAGE with no signal). Fetal placenta displayed Prom chrom at this TSS, a result consistent with the possible function of this region as a tissue-specific promoter.

### 2.7. Myoblast DNA Hypermethylation in Genes That Are Very Highly Expressed in SkM Muscle Tissue

**Figure 6:**
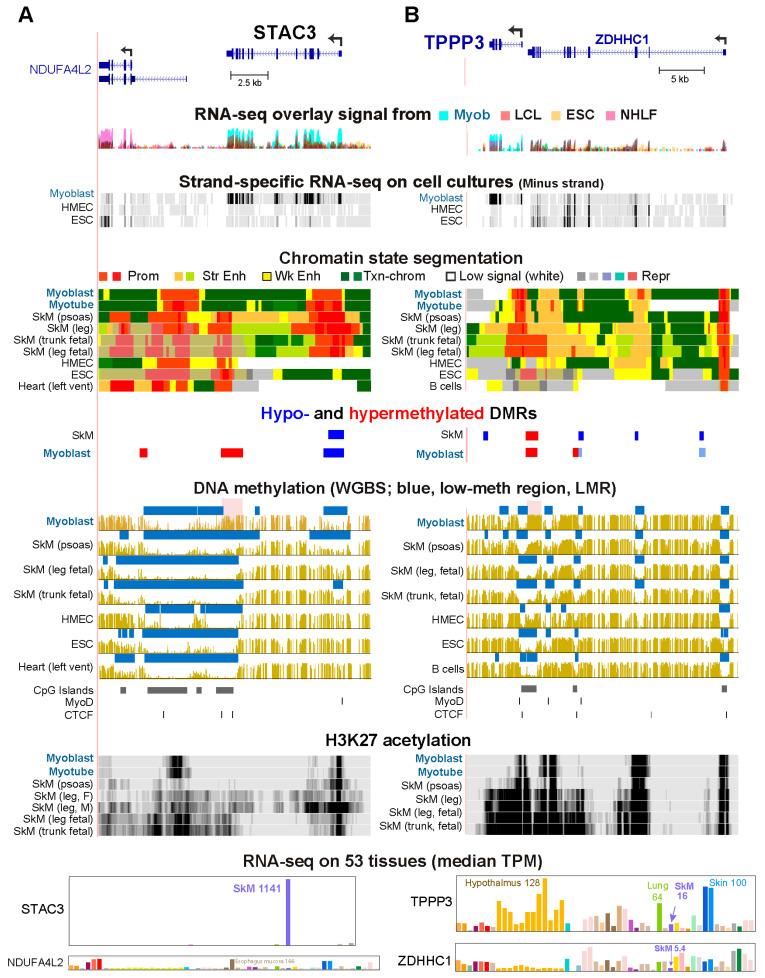
Myob-hyperm DMRs associated with downmodulation of expression in myoblasts vs. postnatal or fetal SkM at *STAC3* and *TPPP3*. (**A**) *STAC3* at chr12:57,628,659–57,646,898. Tissue RNA-seq data for *SHMT2* is included because the 5′ end of this gene overlapped the 3′ end of *NDUFA4L2*, which is included in this figure. (**B**) *TPPP3* at chr16:67,421,154–67,451,819. The light blue bars in the DMR track for *TPPP3* had a methylation difference of only −0.25, unlike the threshold of |0.35| for the other DMRs in the figures. Pink highlighting, Myob-hyperm DMRs described in the text. Labels are like those in the legend for Figure 2 except that the vertical viewing range for H3K27ac was 0–10.

Sixteen Myob-preferential genes linked to Myob-hyperm DMRs were associated with much higher expression in postnatal SkM than in myoblasts (ratio of SkM TPM to myoblast FPKM ≥ 10; Table S1). Four of these genes had a hyperm DMR in myoblasts but not in SkM that overlapped Prom or Enh chrom that was found in SkM but not in myoblasts (*STAC3*, *TNNC2*, *TNNT3*, and *MYH7*). *STAC3* encodes a protein that is a critical component of the coupling apparatus for calcium channels in SkM. It is required for normal SkM contraction and is also implicated in controlling myoblast differentiation [39,52,53]. Mutations in *STAC3* are linked to a type of congenital myopathy [54]. In accord with its prominent role in SkM movement, *STAC3* is expressed at especially high levels in SkM (leg SkM: TPM, 1141) and displays large differences in expression in different types of SkM (UCSC Genome Browser, Muscle De Micheli Muscle Sample Track Settings). The gene was covered by a super-enhancer in one leg SkM sample (Figure 6A, H3K27ac tracks). Like SkM, myoblasts displayed tissue-specific hypomethylation at the *STAC3* promoter but had much less Enh chrom than did SkM. Moreover, myoblasts had a Myob-hyperm DMR at the 3′ end of the gene that shortens a constitutive LMR which covered a cluster of CGIs (Figure 6A, pink highlighting). We propose that this Myob-hyperm DMR helps prevent a SkM super-enhancer from forming in myoblasts. In addition, this Myob-hyperm DMR might downmodulate the use of the overlapping CGI for promiscuous transcription initiation. Three other Myob-preferential genes (*SIX2*, *CRLF1*, and *CDH15*) also had a CGI at a Myob-hyperm DMR at or near the 3′ end of the gene.

*TPPP3*, which encodes a tubulin polymerizing protein expressed in only a few cell/tissue types [55], is an example of a Myob-preferential gene that had much higher expression in fetal SkM than in myoblasts or postnatal SkM (Figure S12). This gene has an unusual 1.7 kb CGI promoter region in myoblasts and psoas SkM because only 0.4 kb of the promoter-region CGI is unmethylated in these cell populations (Figure 6B). In myoblasts and psoas SkM, the rest of the upstream part of the CGI was highly methylated through to the 3′ exons of its adjacent neighbor, *ZDHHC1* (Figure 6B, pink highlighting). *ZDHHC1*, which encodes a protein-cysteine S-palmitoyltransferase implicated in innate immunity [39], is capable of run-through transcription out of frame into *TPPP3* in lung cancer [56]. *ZDHHC1* has lower expression than *TPPP3* in myoblasts and SkM. In general, the levels of expression of these two minus-strand genes are inversely related (Figure 6B and Figure S9). Most cell cultures and tissues were essentially unmethylated in the major portion of this CGI from the 5′ end of *TPPP3* to the 3′ end of *ZDHHC1* and have no or much lower expression of *TPPP3* (Figure 6B and Figure S3B).

Consistent with the much higher expression of *TPPP3* in fetal SkM than in postnatal SkM (both in psoas and gastrocnemius leg muscle, Figure S12), the two fetal SkM samples displayed a super-enhancer that spanned *TPPP3* and the 3′ end of *ZDHHC1* (Figure 6B). The hypermethylation in myoblasts and SkM at most of the CGI in the 5′-*TPPP3*/3′-*ZDHHC1* region was missing in fetal myoblasts. These findings suggest that the Myob/SkM hyperm DMR at *ZDHHC1* helps to prevent the formation of a super-enhancer that spanned *TPPP3* and the 3′ end half of *ZDHHC1.* If such a super-enhancer formed in myoblasts and postnatal SkM, it is strongly predicted to further upregulate their expression of *TPPP3*. This fetal SkM super-enhancer, which covers the 3′ portion of *ZDHHC1* is not associated with a considerable increase in this genes’ expression in prenatal leg or trunk muscle (Figure S12). Fetal SkM was also distinctive in expressing considerable amounts of a *TPPP3* isoform (*ENST00000290942*) that has an additional exon 2 and encodes the same protein as the canonical isoform (Figure S12). Therefore, the DNA methylation status upstream of *TPPP3* might influence *TPPP3* pre-mRNA splicing patterns as well as super-enhancer formation or maintenance.

## 3. Discussion

Our analysis of the detailed epigenetic/transcription relationships for 92 genes that were highly and preferentially expressed in myoblasts (Myob-preferential genes) revealed atypical as well as frequently described associations of DNA hypomethylation or hypermethylation with gene expression. Hypomethylation at the promoter region in myoblasts was much more prominent among the set of Myob-preferential genes than among all genes. This could be only partly attributable to there being a lower percentage of Myob-preferential genes with a CGI at their promoter (40%; Table S1) compared with human genes in general (~70%; [57]). Moreover, an additional ~20% of the Myob-preferential genes displayed myoblast-associated extension of constitutively unmethylated regions at CGI promoters (e.g., Figure 3B, Figures S8 and S9). Many studies have demonstrated that experimentally induced hypermethylation of CGI promoters and even some promoters not overlapping CGIs strongly represses their promoter activity (e.g., [58,59]). Moreover, we previously demonstrated in reporter gene assays that CpG methylation targeted only to the non-CGI promoter of *MYO18B*, one of the Myob-preferential genes, or to the non-CGI promoter of *ZNF556*, a myoblast- and cerebellum-associated gene, strongly represses their promoter activity [17,60].

The width of Prom chrom is usually 1–2 kb [61]. For most of the 46 Myob-preferential genes displaying myoblast hypomethylation at the promoter, the widths of Prom chrom regions were >2.5 kb, and for 33% of them, the widths were 4–8 kb (Table S4). Generally, Prom chrom overlaps more TSS-downstream DNA sequence than upstream sequence [62], as was seen for most of the genes with promoter hypomethylation that we studied (Table S1). Unusually broad H3K4me3 domains at active (H3K27ac-enriched) promoter regions tend to be found in cell-identity and highly expressed genes, and such regions have more topological interactions than regular promoters [25,61,63]. Broad H3K4me3 regions can recruit RNA Pol II more efficiently than standard, shorter promoters and compete better with them for RNA Pol II binding [63]. This is partly through binding of shared transcription cofactors [62]. Myoblast-associated, broad H3K4me3/Prom chrom regions that exhibited myoblast hypomethylation might contribute to high transcription levels in myoblasts for many of the Myob-preferential genes.

**Figure 7:**
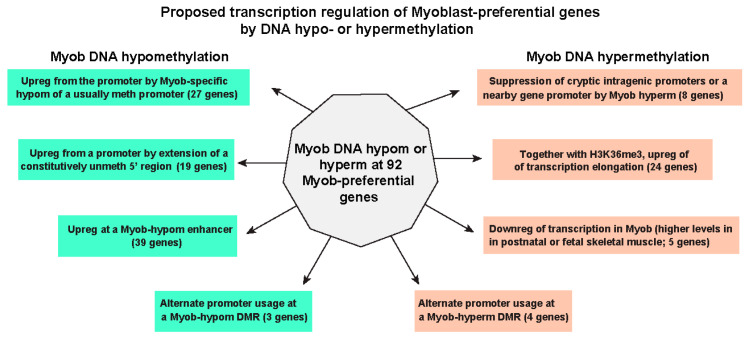
A summary of proposed functions for transcription regulation by myoblast DNA hypomethylation and hypermethylation in Myob-preferential genes. The genes in each category are given in Table S5. Some genes may fit more than one category. Most of the genes in the category of DNA hypermethylation associated with transcription elongation might also be upregulated by suppression of cryptic intragenic promoters outside of CpG islands (Table S5). Myob, myoblast; Myob-preferential genes, genes highly and preferentially expressed in myoblasts. Myob-hypom and Myob-hyperm, hypomethylated or hypermethylated regions in myoblasts vs. six other cell cultures; unmeth, little or no DNA methylation; upreg, upregulation; downreg, downregulation.

The level of DNA methylation at a promoter in a reporter gene construct has been recently shown by Palacios et al. [59] to stably set levels of expression in an analog manner. They used the term “molecular dimmer switch” to describe this type of DNA methylation-induced downmodulation of transcription. In previous studies of myoblast hypermethylation at genes that are expressed at only low-to-moderate levels in myoblasts, we too had concluded that different levels of DNA hypermethylation can sometimes downmodulate, rather than silence, gene expression [9,10,17]. In the current study, we propose a similar DNA hypermethylation/transcription downmodulation relationship for five genes that are highly as well as preferentially expressed in myoblasts (Table S4, Figure 6 and Figure 7). Here, we also provide correlative evidence suggesting that the Myob-associated extension of constitutively unmethylated promoters and DNA hypomethylation at broad H3K4me3 promoter domains of genes that are highly expressed with cell type-specificity can be up-modulatory, probably in an analog fashion. This might involve establishing and/or stabilizing high expression levels in myoblasts by changing the breadth of an unmethylated region rather than the level of methylation at a given DNA segment. However, for promoter regions of Myob-preferential genes that are unmethylated in myoblasts but highly methylated in heterologous cell populations, the DNA hypomethylation might be part of an on-off switch for transcription.

Just as enhancers are essential to differentiation-related expression, Myob-hypomethylated DNA sequences were seen at Enh chrom in almost half of Myob-preferential genes (Figure 7). Often, the Enh segments had only small subregions of hypomethylation that overlapped the strongest peaks of H3K27ac in myoblasts and other strongly expressing cell populations (Figure 3 and Figure 6). Although DNA methylation and histone modifications, including H3K4 and H3K36 methylation, can strongly influence each other’s establishment and function [19,64], they are not redundant [18,59,65,66]. Moreover, DNA methylation/histone modification crosstalk is often context-dependent [18,19,20].

We found many Myob-hypom or Myob-hyperm DMRs in chromatin that lack the canonical histone H3 modifications used by the Roadmap Epigenomics Project (Low signal chrom; Figure 1C,D) [21]. Some Myob-hypom regions that were not embedded in Enh chrom might anticipate and seed the formation of Enh chrom found specifically in SkM [17]. Other myoblast enhancers might use a small subregion with a low CpG density or with no CpGs as an enhancer element (*B3GALT2*, orange square, Figure 5A). Alternatively, genes may utilize a subregion of constitutive unmethylation (*HEYL*, Figure 3B) for binding critical TFs. Previously, we demonstrated in reporter gene assays on an enhancer subregion for the Myob-preferential gene *MYOD1* that hypomethylation of just three of its CpGs in a critical 0.3 kb Core Enhancer element residing in an intergenic myoblast super-enhancer was necessary for its high enhancer activity [67]. The methylation status of the *MyoD1* Core Enhancer in vivo correlates with the gene’s expression in mice and exhibits much of the enhancer activity of the super-enhancer in which it resides [68]. This supports the importance of very localized tissue-specific demethylation for some enhancers.

Contrary to the frequent linking of DNA hypermethylation with gene repression, Myob-hyperm DMRs were found in almost half of the 92 Myob-preferential genes. The strong enrichment of this hypermethylation at Txn-chrom for Myob-preferential genes relative to all Myob DMRs (Figure 1C,D) provides support for the positive correlation of some types of DNA hypermethylation with gene expression [69]. Such hypermethylation is usually in gene bodies [18,21], as we observed among Myob-preferential genes (Figure S2). This intragenic DNA hypermethylation might be partly due to the strong enrichment of H3K36me3 mostly toward the 3′ end of actively transcribed gene bodies [70]. In humans and mice, SETD2/Setd2 catalyzes the methylation of H3K36me2 to form H3K36me3 at transcribed gene bodies and locally recruits DNMT3B/Dnmt3b in previously studied types of mouse and human somatic cells [64,69,71,72]. However, myoblasts have negligible amounts of *DNMT3B* RNA and much higher steady-state levels of *DNMT3A* and *DNMT1* RNAs (average FPKM 0.1, 4.3 and 26, respectively). This suggests the involvement of DNMT3A or both DNMT3A and DNMT1 [73] in gene-body hypermethylation in myoblasts.

Both H3K36me3 and DNA methylation can help suppress intragenic cryptic promoters [70]. In accord with studies of other mammalian cell types [72], we propose that a major function of much of the myoblast hypermethylation within Myob-preferential genes is to prevent misfiring of cryptic/latent intragenic promoters (Figure 7). Some of the intragenic hypermethylation that was not embedded in H3K36me3 chromatin may be silencing latent promoters in the gene body by itself (Figure S2). DNA methylation can function as a primary mechanism for developmentally regulated promoter silencing [71,74], as seen in a small subset of coding genes in normal human tissues [18]. The need for such suppression of latent promoters may be generally greater for highly transcribed, tissue-specific genes. The strong promoter or enhancer activity of Myob-preferential genes and their increased higher-order chromatin interactions at broad H3K4me3 domains [25,61,63] could predispose to misfiring of their latent intragenic promoters. Using reporter gene assays on the intragenic CGI of the *CDH15*, a Myob-preferential gene, we previously demonstrated methylation-induced silencing of promoter activity of this CGI. The intragenic CGI is embedded in Txn-chrom in myoblasts but Prom chrom in most other cell populations [75]. Some nearby gene neighbors of Myob-preferential genes might also need to have their promoter methylated in myoblasts to prevent their deleterious activation in these cells as seen for the neighboring gene-pairs *MUSK/SVEP1* (Figure S5) and *MYOD1*/*KCNC1* [17].

We propose that many of the intragenic Myob-hyperm DMRs in Myob-preferential genes contribute to gene upregulation in cis by positive effects on transcription elongation from intragenic DNA methylation (Figure 7) due not only to the suppression of cryptic promoters but also to preventing formation of repressive chromatin [19,76]. However, in some contexts, intragenic DNA methylation, especially at CpG-rich regions, may slow down transcription elongation, partly because DNA methylation stabilizes the double helix [77]. Another possible role for intragenic DNA methylation could be to help prevent inappropriate interactions of enhancer elements [78]. Alternatively, such DNA hypermethylation at intragenic enhancers could downmodulate expression, as is usually the case for promoters. We found five intragenic or promoter-upstream Myob-hyperm DMRs that were associated with downmodulation of transcription in myoblasts relative to SkM (*STAC3*, *TPPP3*, *MYH7*, *TNNC2*, and *TNNT3*). These Myob-preferential genes were expressed at yet much higher levels in postnatal SkM or fetal SkM relative to myoblasts. They all displayed a super-enhancer overlapping the Myob-hyperm DMR in SkM that was not present in myoblasts. These results support the above-mentioned proposal that Myob-hyperm DMRs can serve as dimmer switches for transcription by preventing formation of super-enhancers in myoblasts that were seen in SkM. Except for the *STAC3* Myob-hyperm DMR at the gene’s 3′ end, these hyperm DMRs were at the upstream or downstream borders of the active Prom chrom region. Therefore, this myoblast hypermethylation could still allow enough promoter activity for high expression of their linked genes but not for the very high expression seen in the few tissues with super-enhancers.

*PITX2*, which is associated with multiple Myob-hyperm DMRs, encodes a TF that has pivotal roles in embryogenesis, including in forming SkM and establishing the normal asymmetrical orientation of many body-cavity organs [45]. It is also implicated in muscle homeostasis, atrial fibrillation, repair of myocardial infarction, and regulation of Na-channel blockers [45,79,80,81]. Transcription levels for this gene must be carefully controlled in vivo because both its overexpression and haploinsufficiency are correlated with disease [45,82]. *PITX2* has Myob-hyperm DMRs overlaying different types of chromatin in myoblasts. This gene is situated in a low methylation valley/canyon in non-expressing cell populations (Figure 5B and Figure S9). Low methylation valleys are frequently associated with developmental genes and broad expanses of repressive H3K27me3 [83], as was seen for *PITX2* in non-expressing cell cultures and tissues. Hyperm DMRs and the loss of some or all H3K27me3 were found in this valley in cell populations that express the gene (myoblasts, SkM, and postnatal skin fibroblasts). The exception was placenta which has the highest expression [84] and had a super-enhancer covering the full length of the gene.

The *PITX2* hyperm DMRs illustrate three likely functions for DNA hypermethylation at a single gene (Table S5). In *PITX2*-expressing cell populations, methylation of the most distal alterative promoter in a region of Low signal chrom was seen, with the exception of placenta, which lacked this methylation. Only placenta displayed Prom chrom at this promoter. This Myob/SkM/postnatal skin fibroblast hyperm DMR might help direct alternative promoter usage away from the most distal promoter without introducing Repr chrom there, which could possibly encroach on the *PITX2A/PITX2B* promoter. The second region of myoblast *PITX2* hypermethylation is at an intragenic CGI that covers mostly weak Prom chrom adjacent to the broad H3K4me3 domain overlapping the *PITX2A/PITX2B* promoter. The hypermethylation at this CGI could prevent it from serving as a promoter in myoblasts. The third region of *PITX2* hypermethylation in myoblasts is immediately downstream of the common 3′ end of the gene. This DMR overlapped the 5′ end of a myoblast-repressed lncRNA gene, *PANCR* (Figure 5B). Myoblast silencing of the neighboring, alternative, or cryptic promoters in these three regions of myoblast hypermethylation may be required because they are close to one of *PITX2*’s two broad H3K4me3 domain/promoters in these cells. For many highly expressed tissue-specific genes, extra protection against unwanted promoter activation in *cis* might be needed due to their possession of promoters with especially high activity on their own or as a result of interactions with very strong enhancers.

Besides providing insights into the epigenetic regulation of Myob-preferential genes in normal cells, our findings may help elucidate the regulation of some of these disease-related genes. *MUSK* is implicated in myasthenia gravis [34,54]. *MUSK*, *RAPSN*, and *STAC3* are linked to congenital muscle disease (including subtypes of congenital myasthenic syndromes), and *PITX2*, *SYNPO2*, and *TPPP3* have been associated with muscle repair, response to stress, or sarcopenia [45,55,85]. Several of the Myob-preferential genes have been implicated in heart dysfunction, including atrial fibrillation, congenital heart abnormalities, and myocardial infarction (*SYNPO2L*, *PITX2*, and *TRIM55*). [42,45,86]. Lastly, many of these genes (*PITX2*, *MUSK*, *SYNPO2*, *TPPP3*, and *STAC3*) have significant associations with cancer [34,45,55,87,88], a disease in which altered DNA methylation is prominently involved causally or as a marker.

### Limitations of Our Study

The myoblast methylomes [17] used in our study were from different batches of cells than those used for chromatin epigenetic and RNA-seq profiles. This might confound comparisons among them. Despite this limitation, we found strong positive associations of our Myob-hypom DMRs with Enh chrom and MyoD binding sites in myoblasts. Moreover, the many expected associations of our Myob DMRs with both overlying histone modifications and transcription profiles of linked genes argue for the reliability of the myoblast DMRs. Our finding that most of the epigenetic and transcription analyses are correlated also partially mitigates possible complications of using RNA-seq levels as a marker for transcription per se and the absence of transcription-regulatory histone modifications not included in the Roadmap Epigenomic Project-determined chromatin states. In addition, these correlations indicate that the cell heterogeneity of tissue methylomes to which we compared myoblast methylomes in individual gene neighborhoods do not affect the conclusions. Although the research in this study is correlative, we have previously published reporter gene assays on three of the Myob-preferential genes (*MYO18B*, *CDH15*, and *MYOD1*) demonstrating that de novo DNA methylation targeted to their promoter, postulated intragenic cryptic promoter, or strongest intergenic enhancer element strongly repressed their promoter or enhancer activity in myoblast host cells [17,60,75]. Moreover, our previous reporter gene studies showing downregulation of transcription in transfected myoblasts by targeted promoter-upstream hypermethylation at *TBX15* and *SIM2* [10,17] serve as models for the proposal of a similar in vivo phenomenon at *TPPP3* and several other Myob-preferential genes analyzed in this study.

## 4. Materials and Methods

### 4.1. Methylomes and DMRs

The methylomes were generated from batches of primary myoblast cell cultures [17] that had been characterized by immunohistochemistry to verify a minimal extent (<10%) of contamination with fibroblast-like cells. The WGBS and EM-seq methylomes were from one and three of these cultures, respectively, as previously described [17]. As previously detailed [17], the DMRs were determined from comparison of myoblast methylomes (WGBS and EM-seq) to publicly available WGBS methylomes [22] from six non-cancer cell cultures, namely, foreskin fibroblasts, adipose-derived mesenchymal stem cells induced to differentiate to adipocytes (ADS-adipocytes), prostate epithelial cells (PrEC), human mammary epithelial cels (HMEC), prenatal lung fibroblasts (IMR90) and human embryonic stem cells (H1 ESC). For DMR determination, we first identified differentially methylated sites (DMS) for the three EM-seq myoblast profiles relative to the six non-myoblast WGBS cell cultures by logistic regression after pooling technical replicates and merging strands, excluding all sites without at least 5× coverage in two or more of the myoblast samples and four or more of the cell cultures. DMRs were derived from DMS using the UP algorithm, which identifies statistically significant clusters of DMS as previously described [89] and filtered to include DMRs for methylation difference. As previously described [17], DMRs were assigned to genes, giving preference first to coding genes over non-coding genes and to proximity to the TSS of genes or overlap with the gene body. Gene vicinity region was exclusively assigned in the following order of precedence: promoter vicinity (TSS −2 to +0.5 kb; TSS +0.5 to +2 kb; TSS −2 to −5 kb), intragenic (TSS +2 kb to transcription end site, TES); gene downstream (TES to TES +2 kb), and intergenic (all other locations). In this study, where we focused on <100 genes instead of the whole genome, we used a filter of differences in methylation of ≤−0.25 for Myob-hypom DMRs and ≥0.25 for Myob-hyperm DMRs instead of ≤−0.35 and ≥0.35 as previously. Figure S1B shows that similar distributions of overlap of Myob DMRs with different chromatin states were obtained with either threshold. However, in the figures, we show only DMRs which met the more stringent criteria that were previously used [17], unless otherwise noted. SkM DMRs had been determined by the same methods as for Myob DMRs, except that the comparisons were of SkM WGBS profiles to those of heart (left ventricle, aorta, lung, adipose tissue (subcutaneous), and monocytes [17].

### 4.2. Bioinformatics

Generally, RefSeq Curated gene isoforms [22] are illustrated in figures. Most bioinformatic profiles, including WGBS profiles for samples other than myoblasts and CGIs, and chromatin state segmentation profiles (18-state model [21]) were from the UCSC Genome Browser using the hg19 (mainly) or hg38 reference genomes [22], as previously described [17,90]. Our slightly modified names for chromatin states [21] described in the figures are as follows: promoter chromatin (Prom, State 1); weak promoter chromatin (States 2 or 4, H3K4me3 with only a low H3K27ac signal); enhancer chromatin (EnhG, States 7 or 8; EnhA, States 9 or 10); mixed enhancer and promoter chromatin (Prom/Enh, State 3); weak enhancer chromatin (State 11); transcription chromatin (Txn-chrom, States 5 or 6); repressed chromatin (Repr, States 12–17); Low signal chromatin (Low signal, State 18). Super-enhancers were determined using the dbSUPER database [24] and confirmed by visual examination of H3K27ac tracks in the UCSC Genome Browser (vertical viewing range, 0–10). SkM (psoas) for WGBS and chromatin state was a mixture of psoas DNA from a 3-y male and a 34-y male; SkM leg was from an unspecified leg muscle. SkM for GTEx data, from gastrocnemius leg muscle. CTCF and MYOD binding to Myob DMRs was determined from the UniBind Permissive 2021 database for human samples [36]. Further details about samples for tracks in the UCSC Genome Browser were given previously [17]. Transcription data was from the following UCSC Browser tracks or hubs: cultured cells (strand-specific RNA-seq, ENCODE/Cold Spring Harbor Lab, or non-strand-specific RNA-seq, Transcription Levels Assayed by RNA-seq on 9 cell lines/ENCODE [26]); GTEx (Genotype-Tissue Expression, medium TPM from RNA-seq from hundreds of samples for each tissue [91]), and the Human Protein Atlas (single-cell RNA-seq [84]). For comparisons of RNA levels in myoblasts and myotubes, we used our previously generated RNA-seq data on primary myoblast cell cultures from our lab [92]. Expression of genes in myoblasts relative to five other types of cell cultures (ESC, HUVEC, NHEK, NHLF, and an LCL) was determined from FPKM data (the above-mentioned ENCODE non-strand-specific RNA-seq [26]). The genes that we analyzed in detail and show in figures had similar chromatin state profiles for myoblasts and myotubes. All illustrated tracks in figures are publicly available at the USCS Genome Browser, except for the DMR tracks and EM-seq on myoblasts. LMRs were determined by the Smith lab [35], and they refer to them as Hypom-methylated regions (HMRs). In addition, for the myoblast WGBS or EM-seq tracks, we previously determined LMRs using the method of the Smith lab [17]. Relative strength of MyoD binding was from ChIP-seq data on C2C12 myoblasts and identification of orthologous human/mouse sequences [93]. For determining overlap of Myob-preferential genes with SkM-preferential genes, we used the criteria of SkM expression at TPM ≥ 5 and the expression ratio of SkM TPM divided by the median of 54 tissue types (Table S1) of ≥5. Gene ontology analysis used the coordinates of all the Myob-preferential genes’ Myob DMRs in the Genomic Regions Enrichment of Annotations Tool (GREAT, http://great.stanford.edu (accessed on 25 November 2025), v 4.0.4 with no background set [27]).

### 4.3. Statistical Methods

Distributions of chromatin types in Myob-hyperm or Myob-hypom DMRs linked to Myob-preferential genes relative to all Myob-hyperm or Myob-hypom DMRs in Figure 1C,D were compared using two-sided tests of proportion for each type, with all *p*-values (Figure 1C) adjusted for multiple comparisons using the Benjamini–Hochberg procedure.

## 5. Conclusions

Our analysis of genes that are highly and preferentially expressed in myoblasts gives new insights into epigenetic/transcription relationships. High transcription levels in many, but not all, of the Myob-preferential genes can be attributed, in part, to super-enhancers and to broad regions of promoter chromatin (broad H3K4me3 domains) overlapping their 5′ ends and containing subregions that are specifically hypomethylated in myoblasts. The Myob-associated promoter hypomethylation can be acting either as part of an on-off switch or as a molecular dimmer switch for transcription initiation. The dimmer switch function is especially likely where the widening of promoter chromatin and of the overlapping region of promoter hypomethylation is found at a constitutively unmethylated CGI/promoter. The intragenic myoblast hypermethylation seen in about half of the Myob-preferential genes may have multiple functions. It might suppress cryptic or alternative promoters, prevent super-enhancer formation, and facilitate transcription elongation. Particularly active promoters and enhancers are associated with more 3D chromatin interactions and strong competition for binding transcription factors compared with standard promoters and enhancers. We propose that genes which are highly and preferentially expressed and which have strongly active, and often wide, promoters or enhancers might have an especially strong need for overlapping or nearby cell type-specific DNA methylation to direct transcription regulation.

## Data Availability

EM-seq and WGBS data for myoblasts have been deposited in the GEO database under accession number GSE287814 (GEO Accession viewer). These data from technical duplicates were pooled for myoblast methylome profiles.

## Supplementary Materials

The following supporting information can be downloaded at: https://www.mdpi.com/article/10.3390/epigenomes10010020/s1, Figure S1: Similar epigenetic profiles for Myob-preferential genes using two methylation difference thresholds; Figure S2: Gene vicinity distribution of DMRs associated with Myob-preferential genes; Figure S3: RNA-seq profiles (ENCODE) from many different types of cell culture for genes in Figure 2, Figure 3, Figure 4, Figure 5 and Figure 6. Figure S4: Single-cell RNA-seq data (Human Protein Atlas) for genes in Figure 2, Figure 3, Figure 4, Figure 5 and Figure 6. Figure S5: *MUSK* is the only gene in its gene neighborhood with myoblast preferential expression and epigenetics; Figure S6: The 5′-end of *TRIM55* and the cluster of three tRNA genes that are its neighbors; Figure S7: Expression of *SYNPO2L* and its upstream gene neighbor, *MYOZ1*, in tissues and cell cultures; Figure S8: *FNDC5*, a gene that lacked a Myob-preferential DMR but had a myoblast- and SkM-associated extension of a constitutive low-methylated region (LMR) at the promoter; Figure S9: *ADAMTS5*, another example of a Myob-preferential gene with extension of constitutive promoter hypomethylation that generally correlated with gene expression levels; Figure S10: *PITX2*: Three regions of Myob-hyperm DMRs are associated with different aspects of gene regulation; Figure S11: Gene neighborhood of *PITX2* including lncRNA gene *PANCR;* Figure S12: *TPPP3* and its neighbor *ZDHHC1*; Table S1: Myoblast-preferential genes: Expression and summary of myoblast hypomethylation or hypermethylation; Table S2: Myoblast DMRs in highly and preferentially expressed myoblast genes: Characteristics of individual DMRs; Table S3: Differences in the type of myoblast chromatin overlapping Myob DMRs for Myob-preferential genes vs. all Myob DMRs; Table S4: Genes whose myoblast differential methylation fits proposed functional categories; Table S5: Low-methylated regions (LMRs) seen preferentially in myoblasts for genes without myoblast-hypomethylated DMRs.

## Abbreviations

The following abbreviations are used in this manuscript; less frequent abbreviations are defined in the text or Figure legends:

Ac	acetylation
CAGE	5′ cap analysis of gene expression
CGI	CpG island
Chrom	chromatin
ESC	embryonic stem cells
Enh	enhancer
H3	histone H3
IMR90	fetal lung fibroblasts
LCL	lymphoblastoid cell line
Me	methyl
Myob-hypom DMR	myoblast hypomethylated differentially methylated region
Myob-hyperm DMR	myoblast hypermethylated differentially methylated region
NHEK	human epidermal keratinocytes
NHLF	human lung fibroblasts
Prom	promoter
Repr	repressed
SkM	skeletal muscle
TSS	transcription start site
Txn-chrom	transcription-type chromatin (H3K36me3-enriched)
