# Supplementary material for: Epigenetics of Genes Displaying High and Preferential Expression in Myoblasts
Source: Epigenomes. 2026 Mar 13;10(1):20. doi: 10.3390/epigenomes10010020 (PMC13025873; doi:10.3390/epigenomes10010020)
Supplement: Supplementary file 1 [file epigenomes-10-00020-s001.zip › EhrlichK Suppl Figures_Epigenetics of genes highly & preferentially expressed in myoblasts RevisedF 2_19_26.pdf]

Supplemental figures S1 to S12

Epigenetics of genes displaying high and preferential expression in myoblasts

Kenneth C. Ehrlich, Michelle Lacey, Sriharsa Pradhan, and Melanie Ehrlich

Figure S1: Similar epigenetic profiles for Myob-preferential genes using two methylation difference thresholds

Figure S2: Gene vicinity distribution of DMRs associated with Myob-preferential genes

Figure S3: RNA-seq profiles (ENCODE) from many different types of cell culture for genes in Figures 2 - 4.

Figure S4: Single cell RNA-seq data (Human Protein Atlas) for genes in Figures 2 - 6.

Figure S5: *MUSK* is the only gene in its gene neighborhood with myoblast preferential expression and epigenetics

Figure S6: The 5'-end of *TRIM55* and the cluster of three tRNA genes that are its neighbors

Figure S7: Expression of *SYNPO2L* and its upstream gene neighbor, *MYOZ1,* in tissues and cell cultures

Figure S8: *FNDC5,* a gene that lacked a Myob-preferential DMR but had a myoblast- and SkM-associated extension of a constitutive low-methylated region (LMR) at the promoter

Figure S9: *ADAMTS5*, another example of a Myob-preferential gene with extension of constitutive promoter hypomethylation that generally correlated with gene expression levels

Figure S10: *PITX2*: Three regions of Myob-hyperm DMRs are associated with different aspects of gene regulation

Figure S11: Gene neighborhood of *PITX2* including lncRNA gene *PANCR*

Figure S12: *TPPP3* and its neighbor *ZDHHC1*

A

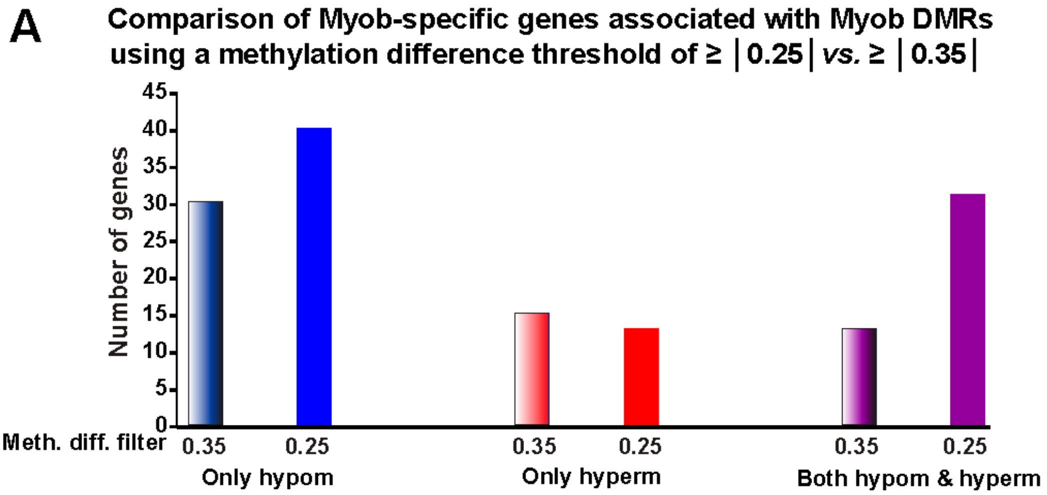

B

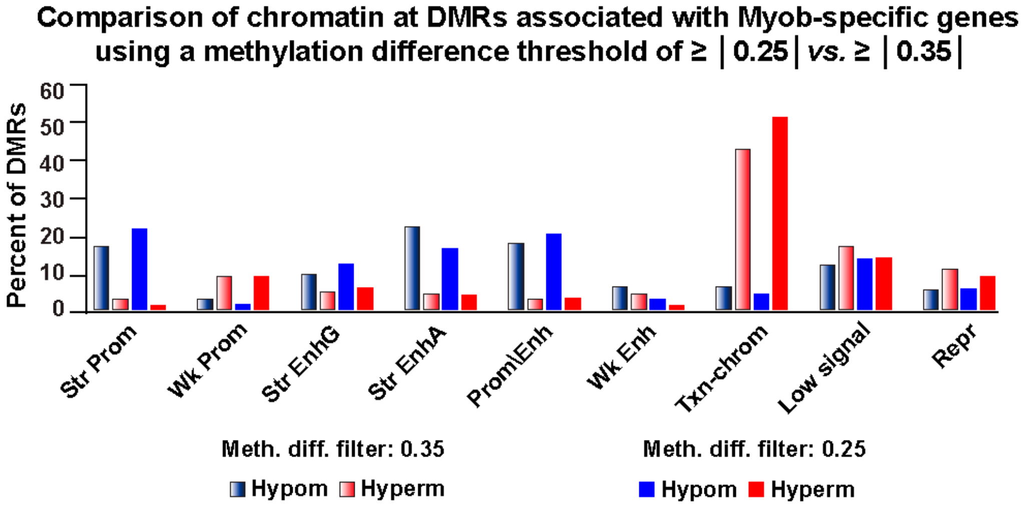

Figure S1. **Similar epigenetic profiles for Myob-preferential genes using two methylation difference thresholds**. (**A**) Number of Myob DMR-associated genes when the absolute value for the methylation difference threshold is varied from 0.25 to 0.35. (**B**) The overlap of Myob DMRs (percent of DMRs) with a given chromatin state (Roadmap Epigenomics Project, 18-state model) when the methylation difference threshold was varied as in Panel A. This figure is related to Figure 1A – D, in which the absolute value of the threshold for methylation difference was 0.25. Myob-preferential genes, coding genes that are highly and selectively expressed in myoblasts (see Results). Meth. Diff filter, the absolute value of the threshold of methylation difference for determining DMRs; hypom, hypomethylated; hyperm, hypermethylated; Str Prom, strong promoter chromatin (State 1); Wk Prom, weak promoter chromatin (State 2 or 4); Str EnhG, strong enhancer chromatin enriched in H3K36me3 (State 7 or 8), Str EnhA, strong enhancer chromatin not enriched in H3K36me3 (State 9 or 10), Wk Enh, enriched in H3K4me1 with little or no H3K27ac; Txn, transcription-chromatin (enriched in H3K36me3; State 5 or 6); Repr, repressed chromatin enriched in H3K27me3 or H3K9me3 (State 12 – 17); Low signal, low signal for six H3 markers (State 18).

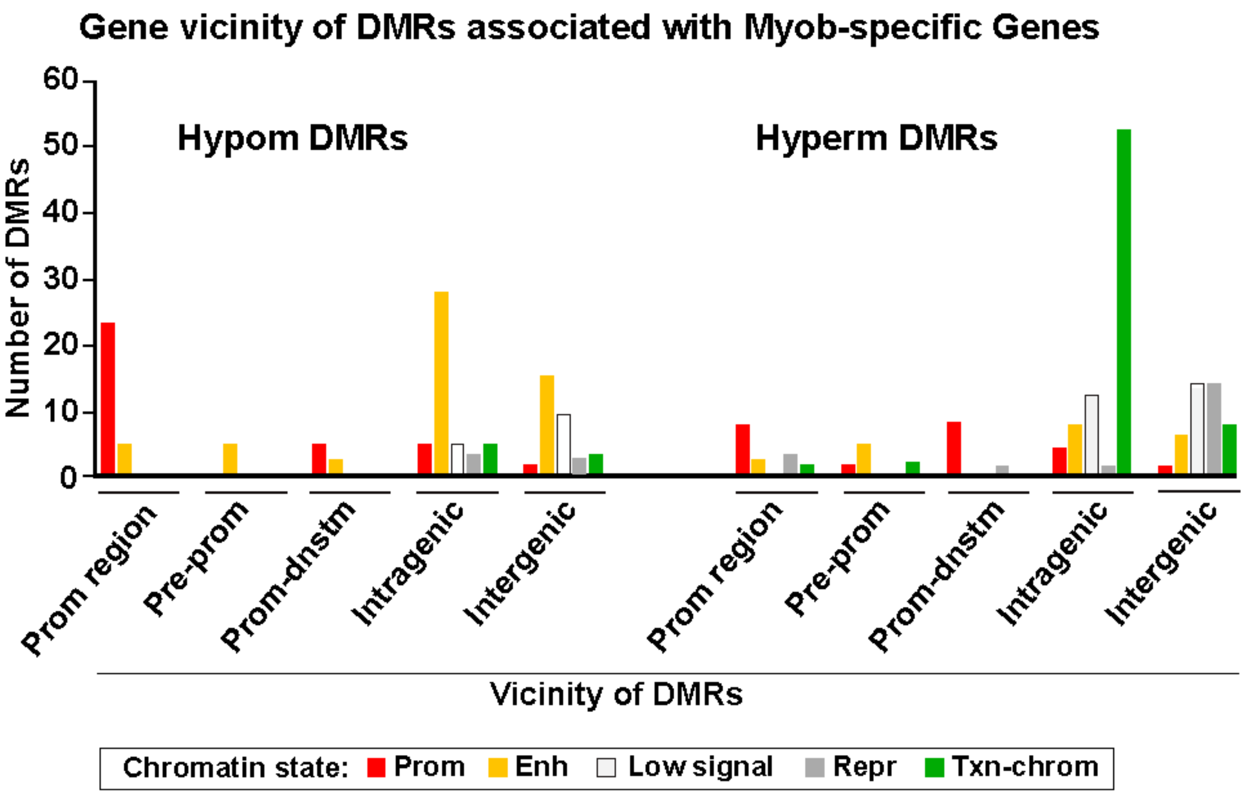

Figure S2. **Gene vicinity distribution of DMRs associated with Myob-preferential genes.** Vicinity is defined with respect to the DMR-assigned coding gene's RefSeq Select TSS except when the gene had an alternative promoter that was used in myoblasts and was not the RefSeq Select TSS (see Table S2, column AB). Prom region, TSS -2 kb to +0.5 kb; Pre-prom, TSS -5kb to -2 kb; Promoter dnstrm (downstream), TSS 0.5kb to 2 kb; Intragenic, TSS +2 kb to 2kb downstream of the transcription end site (TES); Intergenic, all other regions. Chromatin states are as in the legend to Figure S1 with the following simplifications: Prom chrom, States 1, 2, and 4, except for Prom region where State 3 (mixed Prom/Enh) was included; Enh, States 7 - 11, except for regions other than the Prom region where State 3 was included. This figure does not include Myob-associated LMRs or extensions of hypomethylation at CGI promoters that were associated with myoblasts and did not meet the definition of a Myob-hypom DMR (Tables S1 and S4). The number of Myob-hyperm DMRs at intragenic locations in Txn-chrom is skewed upwards by *MYO18B* having 19 such DMRs in its large gene body

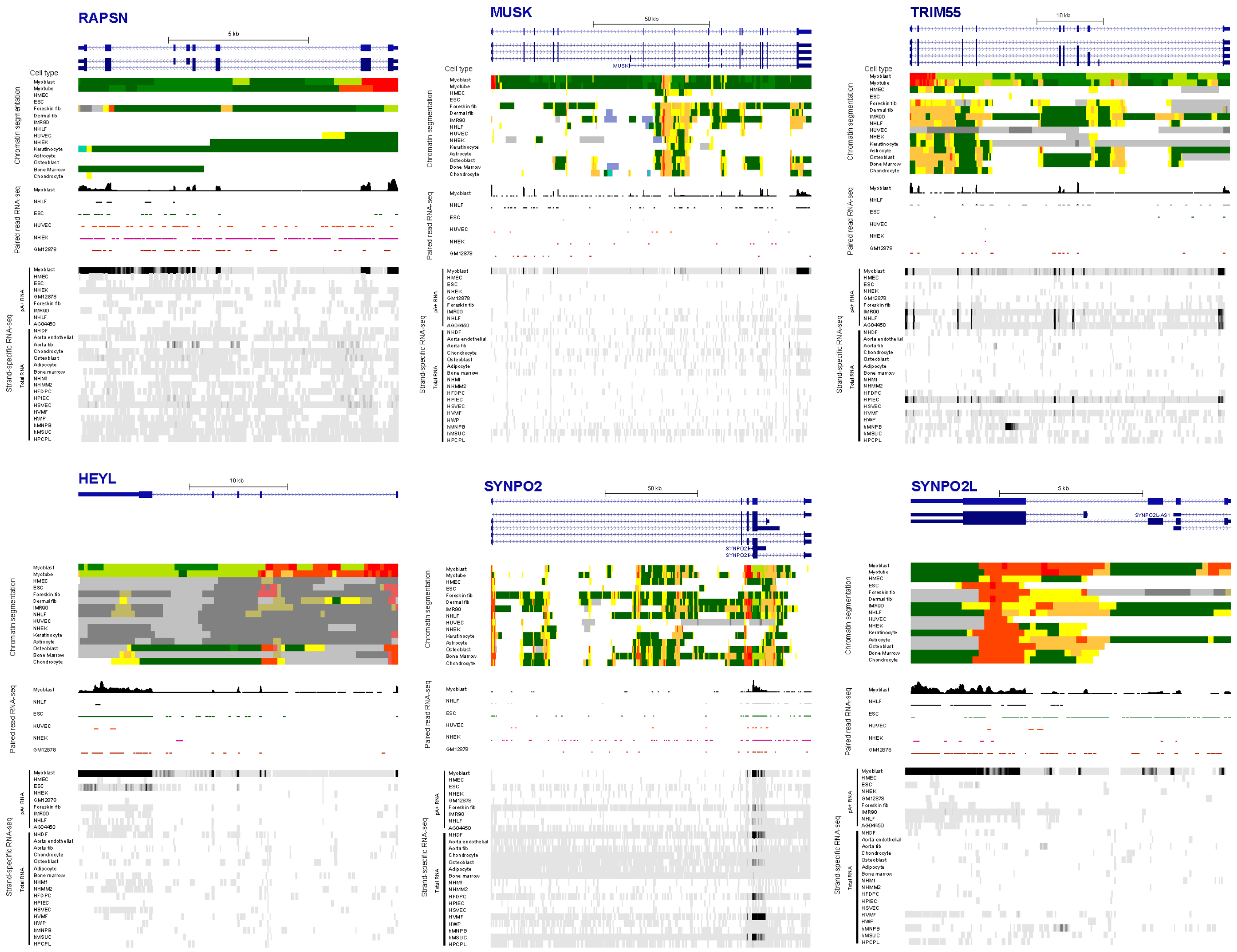

Figure S3A. RNA-seq profiles (ENCODE) from many different types of cell culture for genes shown in Figures 2 - 4. RefSeq Curated isoforms centered on the RefSeq Select isoform. Chromatin state segmentation as in Figures 2 – 4 (Con. By Assay chromHMM Track Settings, Roadmap Epigenomics Project, red, Prom chrom; orange or yellow-green, Enh chrom; green, Txn-chrom; gray, Repr chrom) are shown followed by non-strand-specific RNA seq (ENCODE Regulation Transcription Track Settings, CalTech), and then strand-specific RNA-seq (ENC RNA-seq CSHL Long RNA-seq Track Settings, Cold Spring Harbor Lab) from the UCSC Genome Browser (hg19) are shown. Only the expressed gene for each gene is depicted.. Vertical viewing ranges for non-strand-specific RNA-seq are 0 – 8 and log transformed log (ln(1+x)). For strand-specific RNA-seq they are 0-300 for *TRIM55, PITX2, B3GALT2, TPPP3,* and *STAC3*; 0-1000 for *SYNPO2*; and 0-100 for *RAPSN, MUSK, HEYL,* and *SYNPO2L* with no transformation of data points. Samples for RNA-seq are as follows: GM12878, B-lymphoblastoid cell line; ESC, embryonic stem cells; HUVEC, umbilical vein endothelial cells; myoblasts; NHEK, epidermal keratinocytes; NHLF, lung fibroblast; IMR90, fetal lung fibroblasts; AG04450, fetal lung fibroblasts; BJ, foreskin fibroblasts; aorta fib/HAoAF, aortic adventitial fibroblasts; aorta endothelial/HAoEC, thoracic aortal endothelial cells; chondrocytes/HCH, knee joint chondrocytes; HFDPC, dermal papilla cells; HMEC, mammary epithelial cells; HMEpC, marrary epithelial cells; hMNC-PB, peripheral blood mononuclear cells; adipocytes/hMSC-AT, abdomen adipose from subcutaneous mesenchymal stem cells; bone marrow/hMSC-BM, bone marrow from femoral mesenchymal stem cells; hMSUC, umbilical cord mesenchymal stem cells; osteoblasts/HOB, osteoblasts from femoral head; HPCPL, pericytes from placenta; HPIEpC, epithelial cells from placenta; HSaVEC, saphenous vein endothelial cells; HSMM, myoblasts; HVMF, connective tissue villous mesenchymal fibroblasts; HWP, preadipocytes; NHDF, dermal fibroblasts; NHEK; NHMf, foreskin epidermal melanocytes; NHMM2, adult epidermal melanocytes.

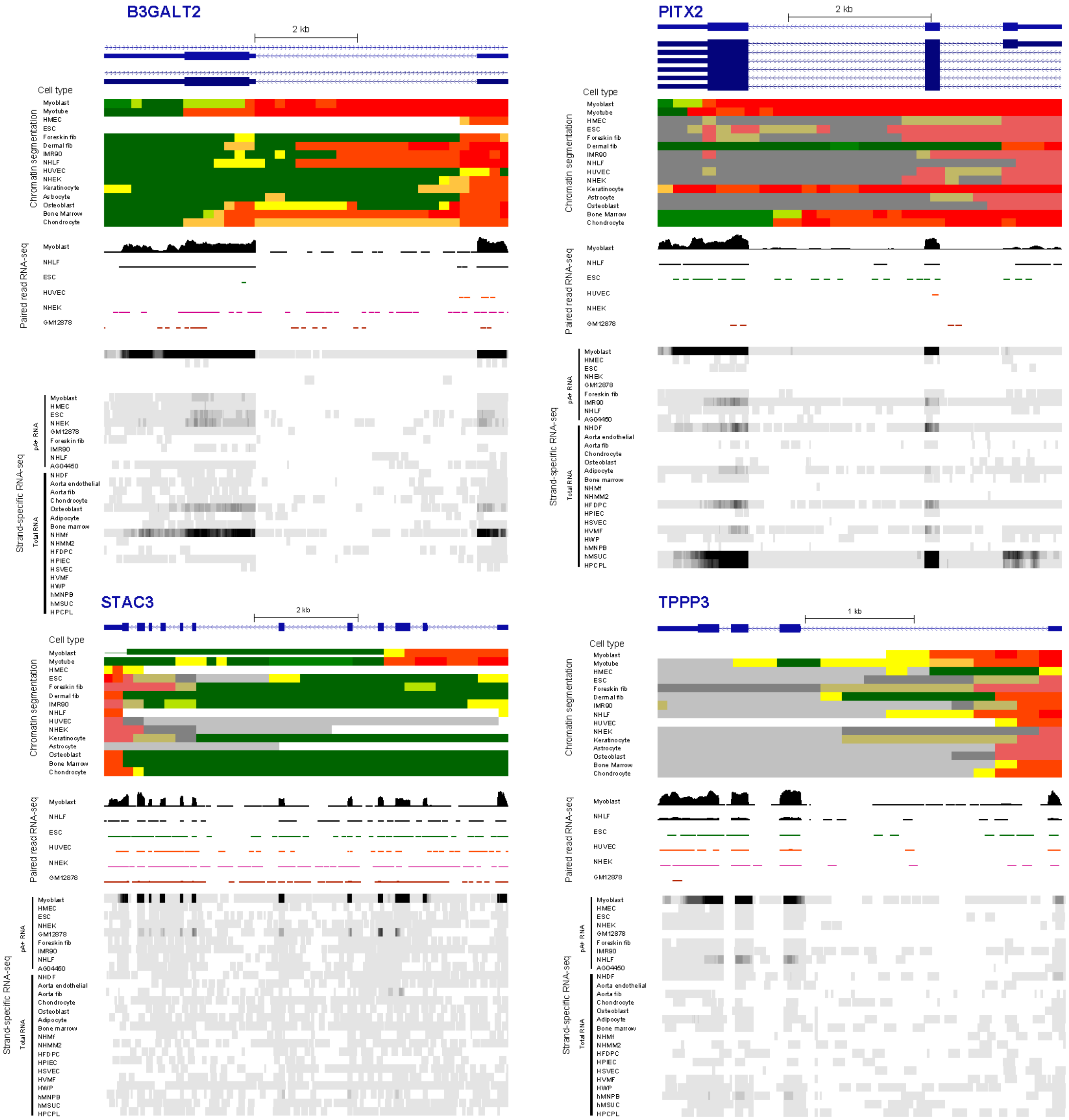

Figure S3B. **RNA-seq profiles (ENCODE) from many different types of cell culture for genes in Figures 5 and 6.** See legend to Figure 3A.

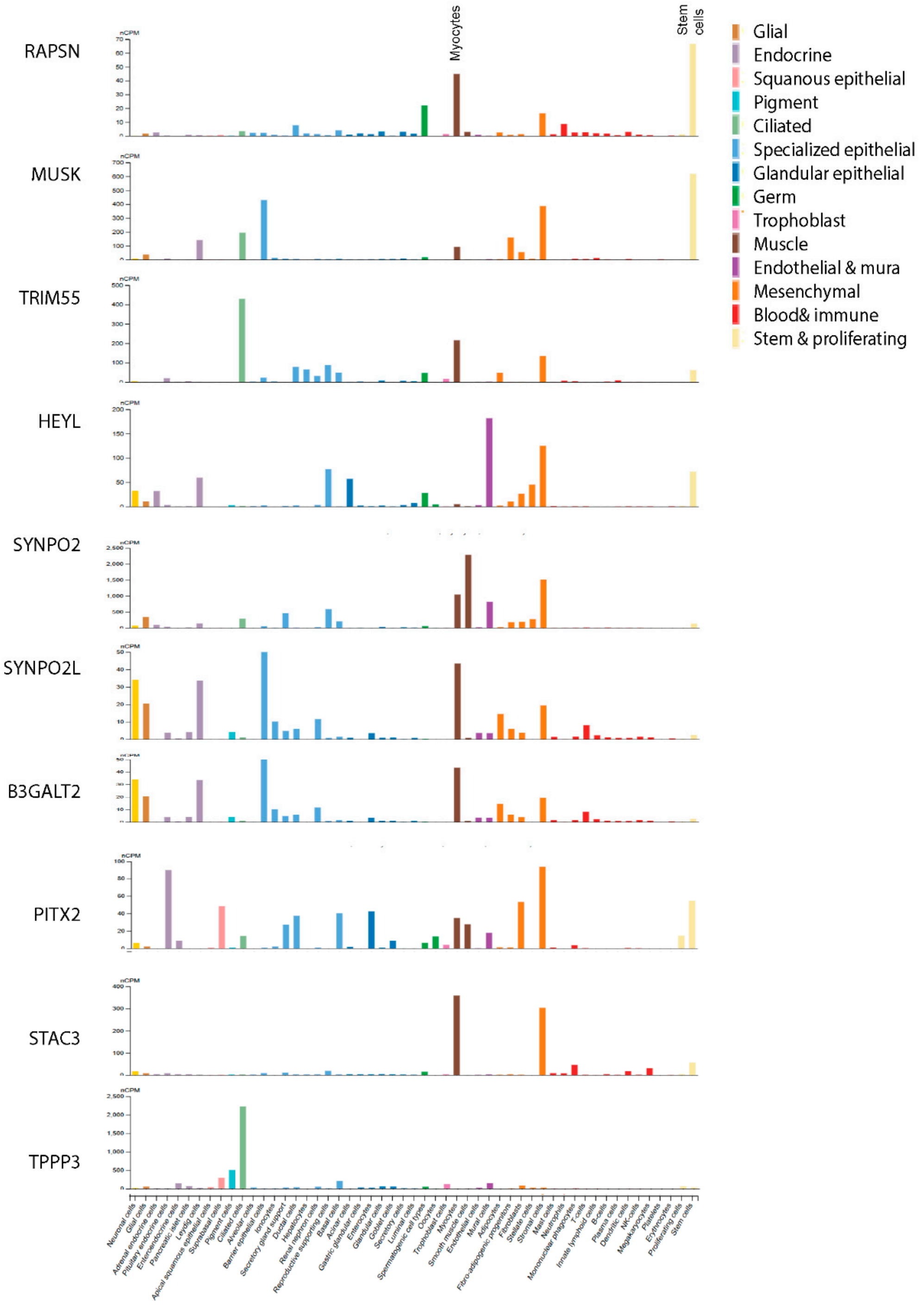

Figure S4. Single cell RNA-seq data (Human Protein Atlas) for genes in Figures 2 - 6. The compiled data (https://www.proteinatlas.org) is from single cell RNA-seq (scRNA-seq) profiles from 154 cell types derived from 34 human tissues. The bar graphs show bulk scRNA-seq values as nCPM (pseudobulked cluster data into counts per million normalized for technical bias by calculating scaling factors based on the expression of shared genes between clusters). The highest orange bar is stromal cells predominantly or only from thymus, which are likely to be myoid cells. The last yellow bar is stem cells, which were predominantly myosatellite cells from skeletal muscle or tongue. For PITX2, the highest expression among an individual cell is in placental fibroblasts (10002 nCPM, not shown) within the fibroblast category, which is shown above.

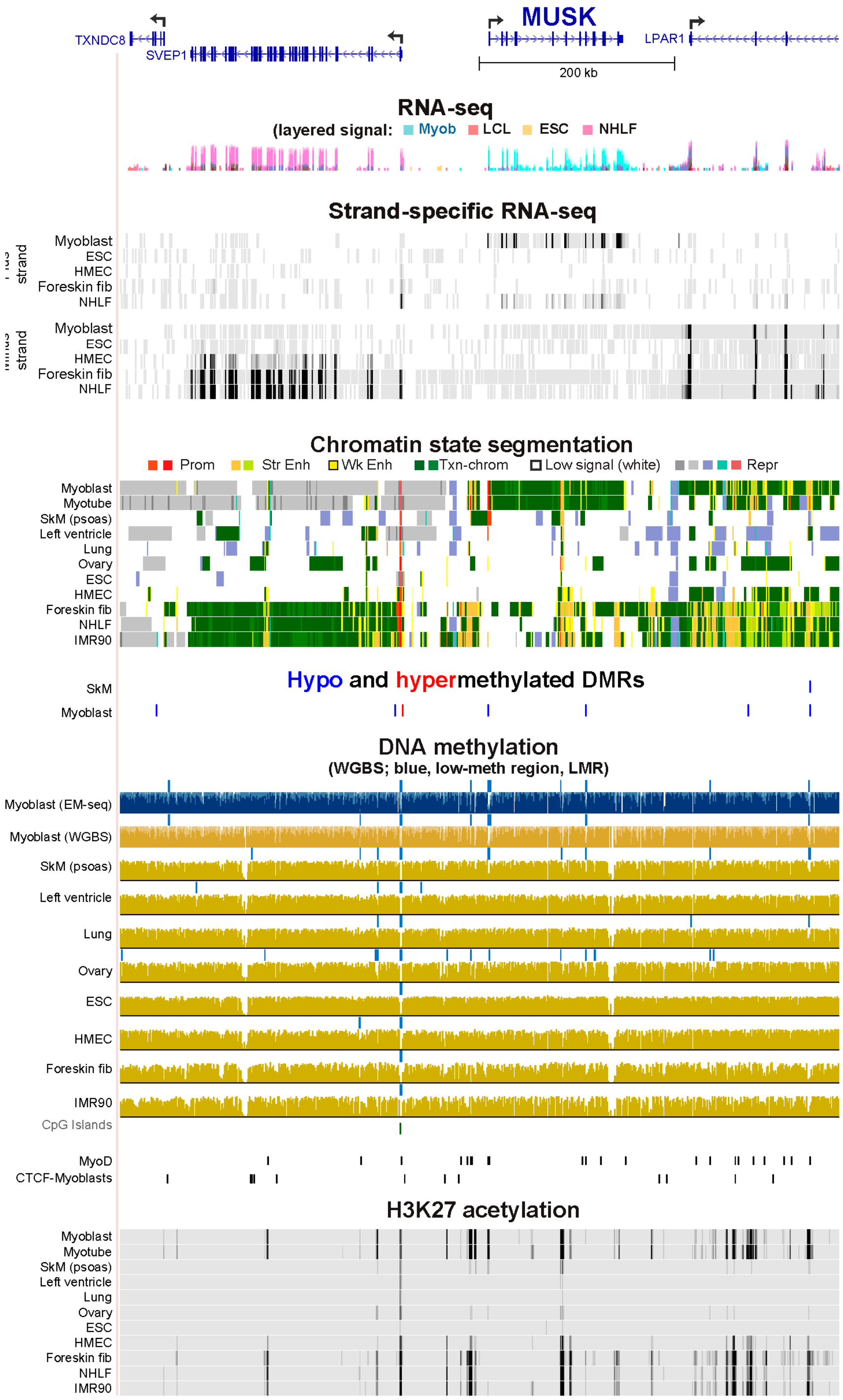

Figure S5. ***MUSK*** **is the only gene in its gene neighborhood with myoblast preferential expression and epigenetics.** The region shown is chr9:113,055,659-113,788,632, hg19, including MUSK gene neighbors *LPAR1, SVEP1,* and *TXNDC8*. Vertical viewing ranges for strand-specific RNA-seq, 0 - 30 and for H3K27 acetylation, 0 - 10. The MyoD and CTCF sites bound in myoblasts are from Unibind. UCSC Genome Browser tracks are labeled as in Figure 2. Abbreviations for this and other gene figures are: fib, fibroblast; SkM, skeletal muscle; HMEC, human mammary epithelial cells; ESC, H1 embryonic stem cells; NHLF, normal human lung fibroblast cell strain; WGBS, whole genome bisulfite sequencing; IMR90, fetal lung fibroblasts; LMR, low methylated region. All tracks are from the UCSC genome browser. This figure complements Figure 2B.

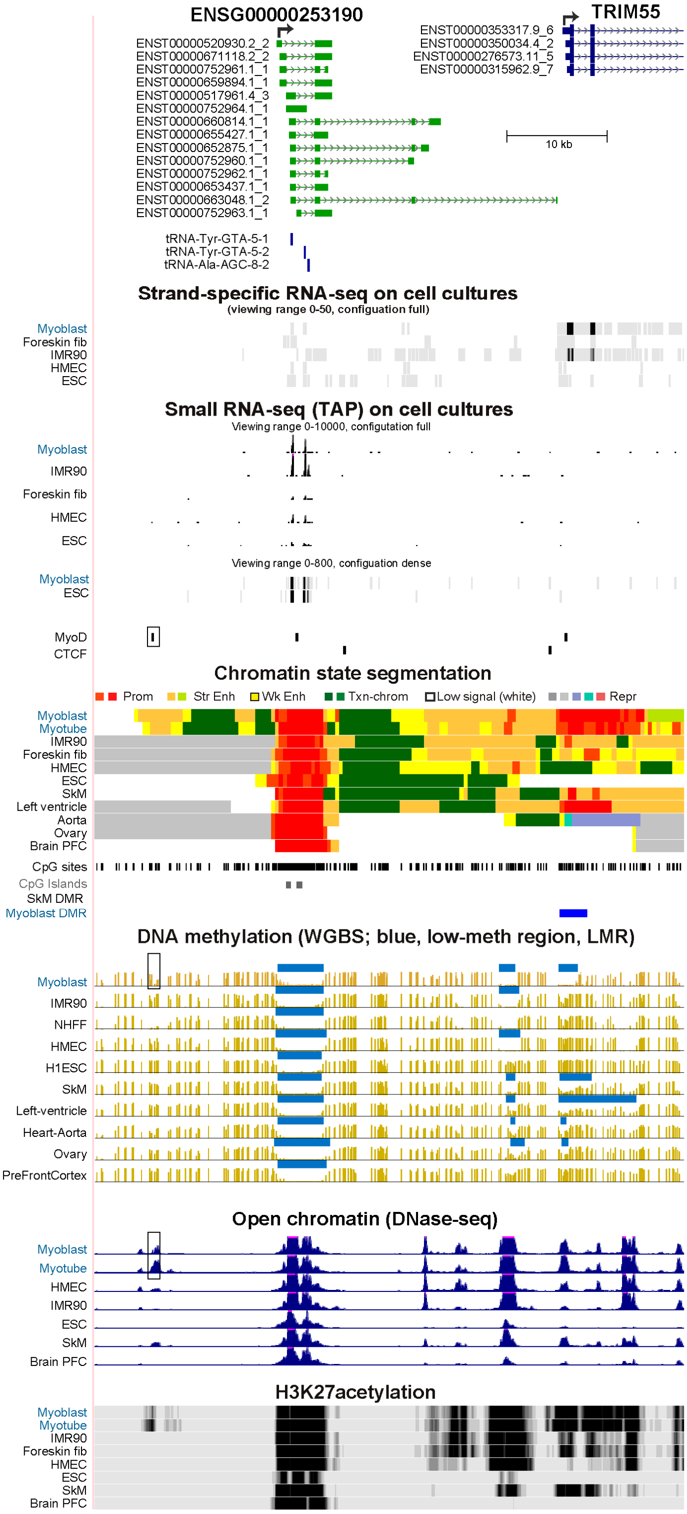

Figure S6. The 5'-end of *TRIM55* and the cluster of three tRNA genes that are its neighbors. The region shown is chr8:67,015,801-67,045,154, hg19 and contains tRNA-Tyr-GTA-5-1, tRNA-Tyr-GTA-5-2, and tRNA-Tyr-AGC-8-2 as well as *TRIM55*. Vertical viewing ranges: strand-specific RNA-seq and large and small RNA-seq, plus strand, 0 - 400; H3K27 acetylation, 0 - 10, DNA-Seq, 0 – 40. Gray boxes, the region of a myoblast/myotube MyoD$^+$ DNaseI-hypersensitive site. In the H3K27ac profile there is evidence for a nucleosome-free region at this region in myoblasts and myotube (not shown). This figure complements Figure 3A.

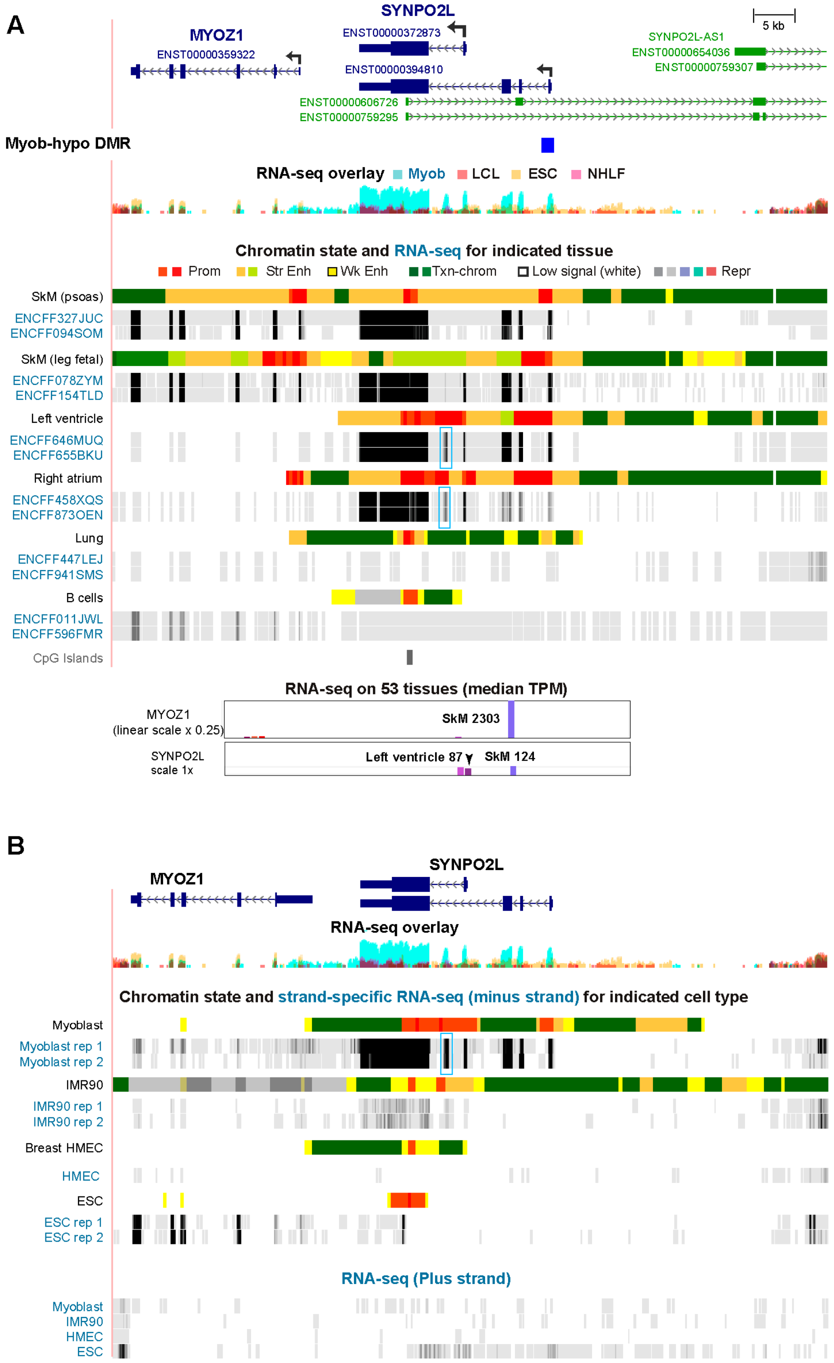

Figure S7. **Expression of *SYNPO2L* and its upstream gene neighbor, *MYOZ1,* in tissues and cell cultures.** *MYOZ1* (*Myozenin 1*) helps tether calcineurin to the sarcomere of cardiac and skeletal muscle. (**A**) Roadmap RNA-seq tracks (only minus-strand profiles are shown; vertical viewing range, 0 – 1) for a given tissue are beneath the analogous chromatin state profile at chr10:73,630,513-73,671,945 (hg38). Biological duplicates are shown. (**B**) ENCODE strand-specific RNA-seq for just the minus-strand at chr10:75390271-75431703 (hg19) (liftover positions to those in panel A) so that cell culture strand specific RNA-seq data can be shown. Vertical viewing range for RNA-seq, 0-30 with technical duplicates when available. GENCODE Ensembl 114 Comprehensive isoforms are shown for *SYNPO2L* because they fit the RNA-seq and chromatin state data best. However, the blue-boxed exon in myoblasts, left ventricle and right atrium was not included in RefSeq or Ensembl isoforms and is seen also in the non-strand-specific myoblast RNA-seq overlay profile. ENST00000394810.3 is analogous to the RefSeq Select isoform for *SYNPO2L*. This figure complements Figure 4B.

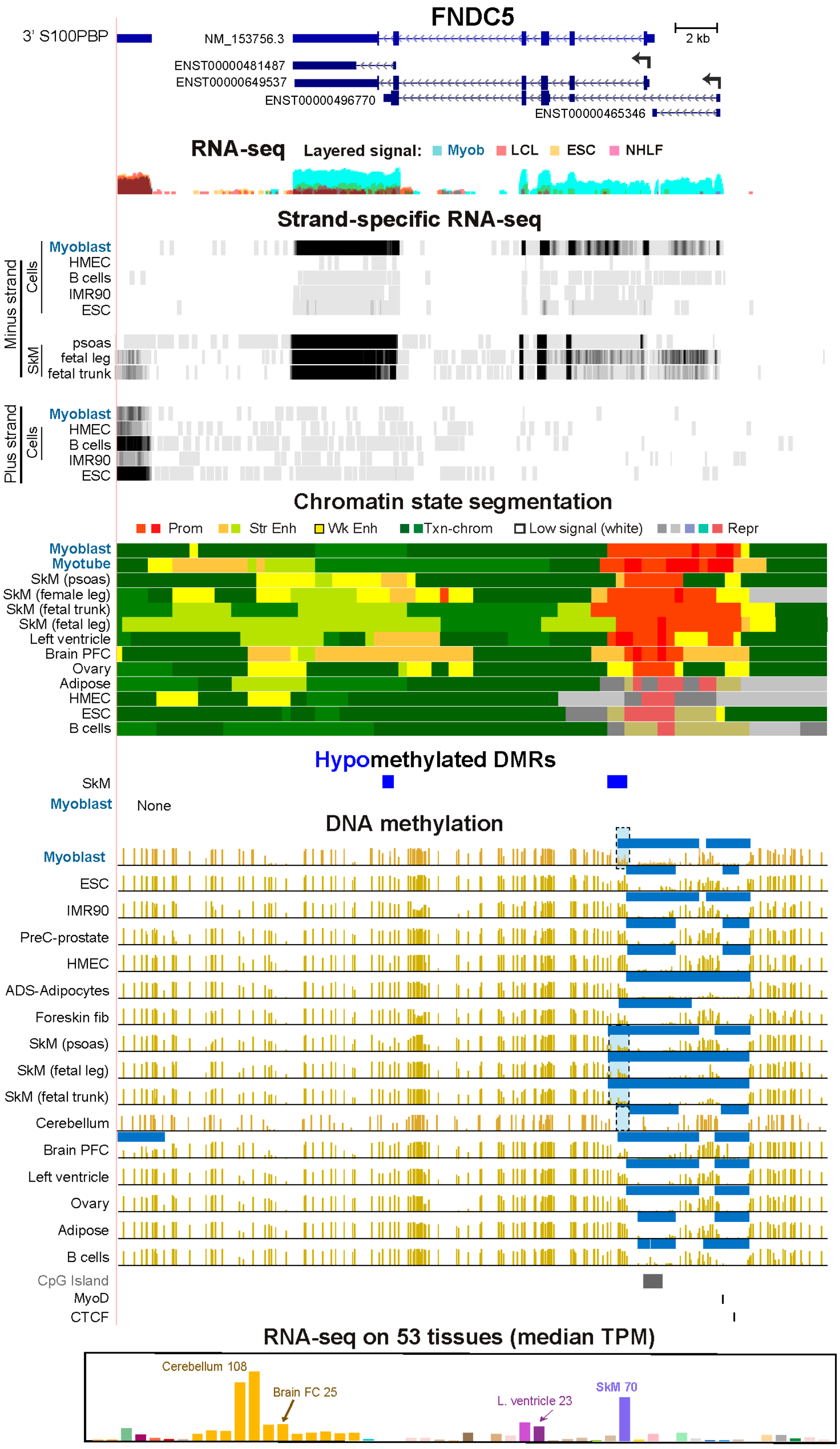

Figure S8. ***FNDC5*, a gene that lacked a Myob-preferential DMR but had a myoblast- and SkM-associated extension of a constitutive low-methylated region (LMR) at the promoter.** *FNDC5* (*Fibronectin Type III Domain Containing 5*) at chr1:33,323,666-33,340,636 (hg19) except for the analogous positions of chr1:32858065-32875035 (hg38) for strand-specific RNA-seq for SkM (Roadmap). The RefSeq Select isoform NM_153756.3 best matches the major signal for myoblasts and postnatal SkM (psoas). Strand-specific RNA-seq for cell cultures used the vertical scale settings of 0-400 for the plus strand and 0-200 for minus strand and for tissues 0-1. The dotted rectangles with light blue highlighting show the regions of the extended LMR in myoblasts and other *FNDC5* high-expressing cell populations.

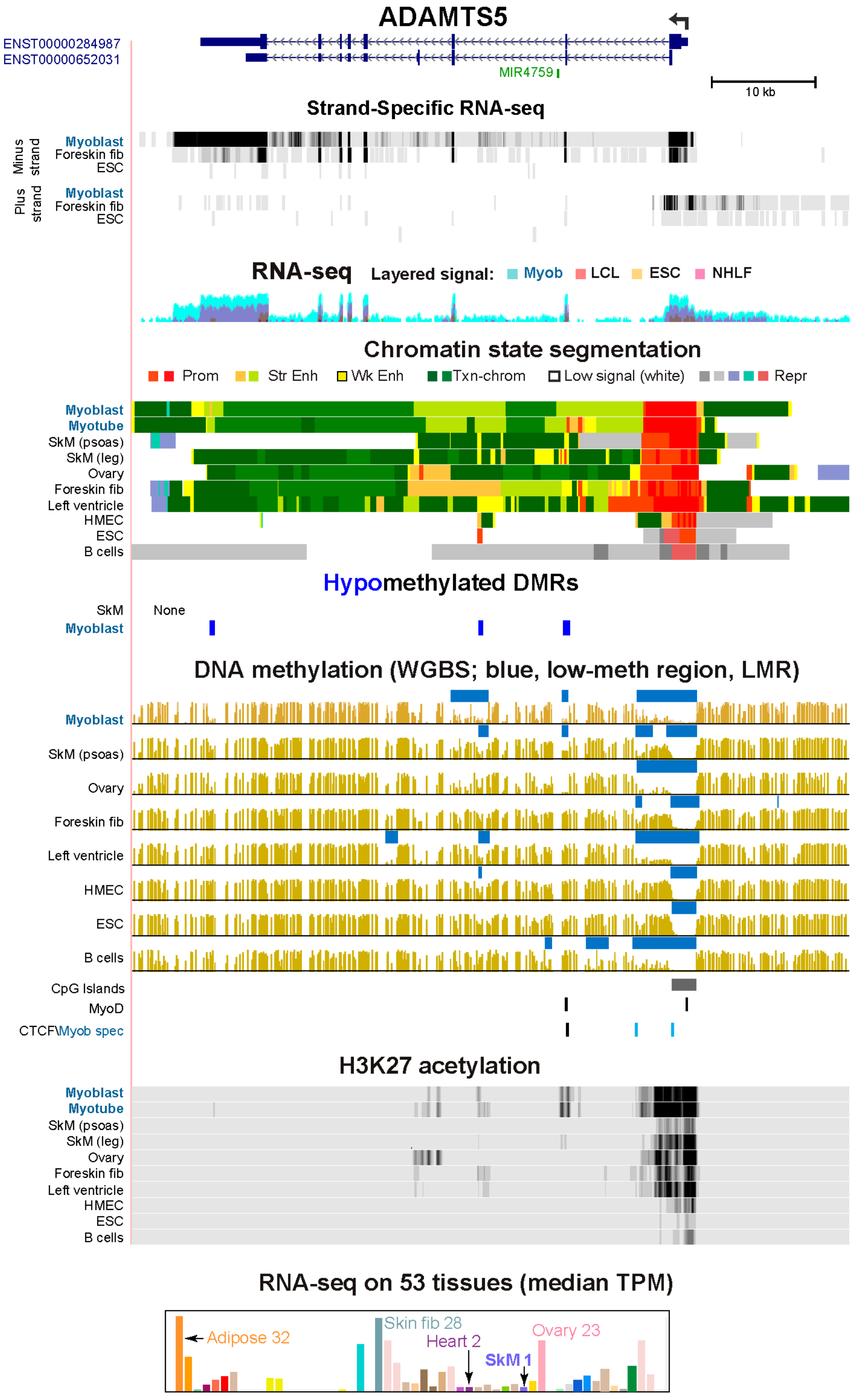

Figure S9. ***ADAMTS5*, another example of a Myob-preferential gene with extension of constitutive promoter hypomethylation that generally correlated with gene expression levels.** *ADAMTS5* (*ADAM metallopeptidase with thrombospondin type 1 motif 5*) at chr21:28,283,297-28,355,690, hg19. Vertical viewing range settings: strand-specific RNA-seq, 0 – 50; non-strand-specific RNA-seq, log scale, 0 – 8; H3K27 acetylation signal, vertical viewing range, 0 – 2. Comparison of these WGBS, chromatin state, and RNA-seq profiles as well as those of 10 other normal cell cultures or tissues indicate that 3' extension of the unmethylated region at the promoter approximately correlated with higher transcription. B cells, shown in this figure, were an exception probably because extensive H3K27me3 in the promoter region and gene body was apparently silencing the gene.

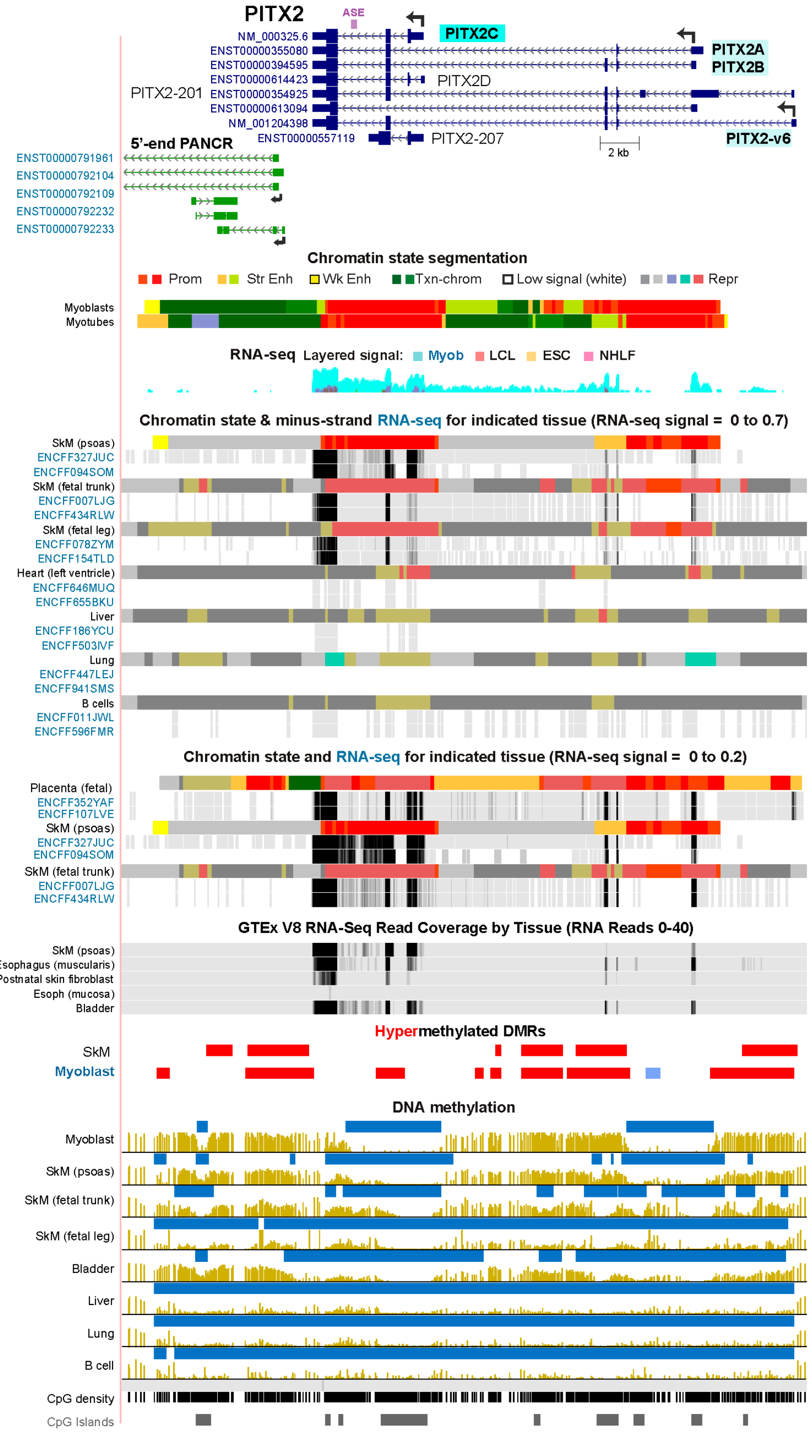

Figure S10. ***PITX2*: Three regions of Myob-hyperm DMRs are associated with different aspects of gene regulation.** *PITX2* (*Paired Like Homeodomain 2*) at chr4:110,607,678-110,642,631 (hg38). NM_00325.6, the RefSeq Select isoform. Roadmap RNA-seq tracks beneath the corresponding chromatin state track are shown. Vertical viewing ranges for RNA-seq are given in the figure. ASE, asymmetry enhancer and the main alternative promoters (highlighted) are described in the text. Light blue box in Myob DMR track, DMR with a methylation difference of only -0.25, unlike the methylation difference threshold of |0.35| for the other DMRs in the figures. This figure complements Figure 5B.

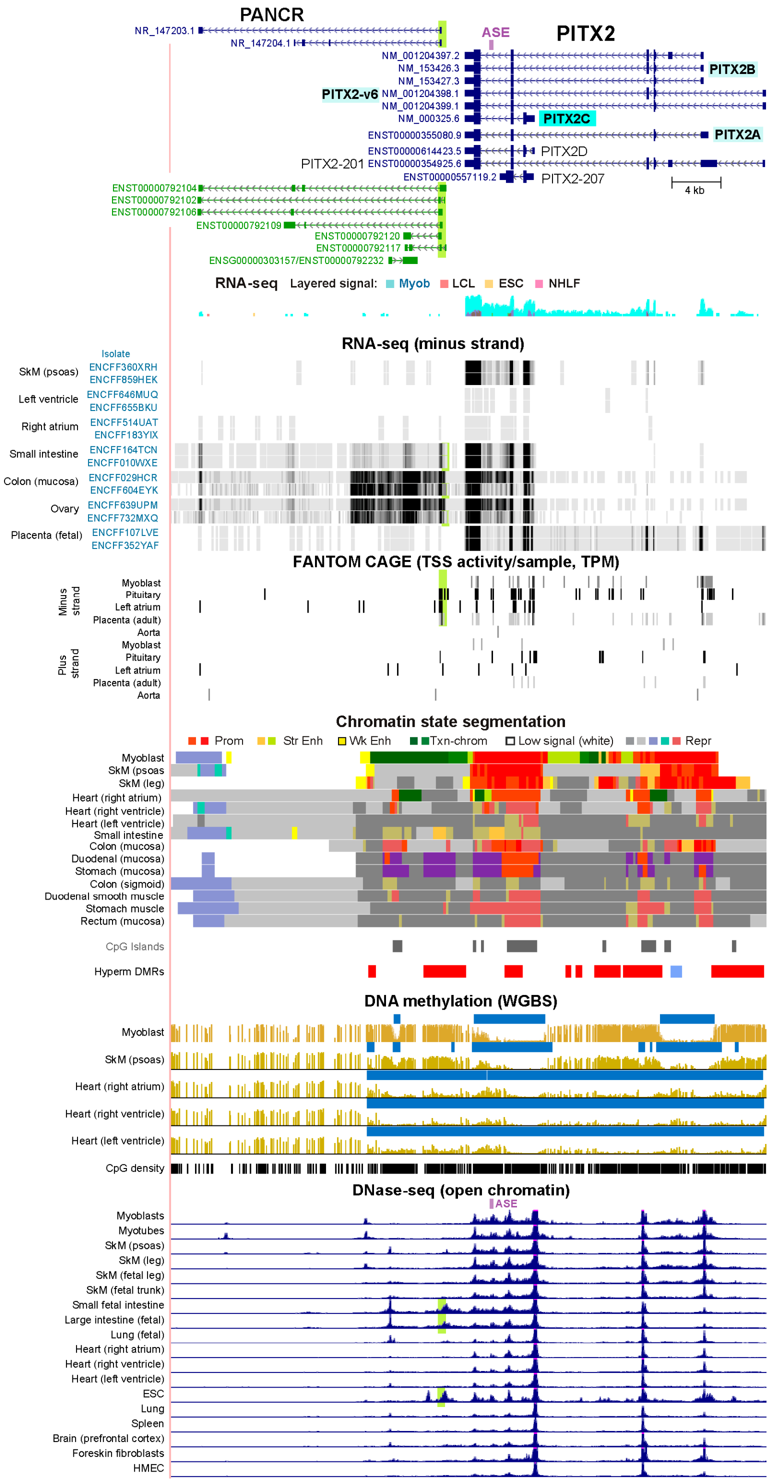

Figure S11. Gene neighborhood of *PITX2* including lncRNA gene *PANCR*. The region shown is chr4:110,593,153-110,642,188 (hg38). The close *PITX2*-downstream gene *PANCR* is a non-coding gene that is expressed most highly in pancreas and with undetectable expression in SkM (see Figure 5B). Vertical viewing range, Roadmap RNA-seq, 0-1; FANTOM CAGE, 0 – 0.2. ESC cells also had a signal for CAGE at the TSS for *PANCR* and displayed bivalent promoter chromatin in that region (not shown). Green highlighting, the 5’ end of *PANCR*.

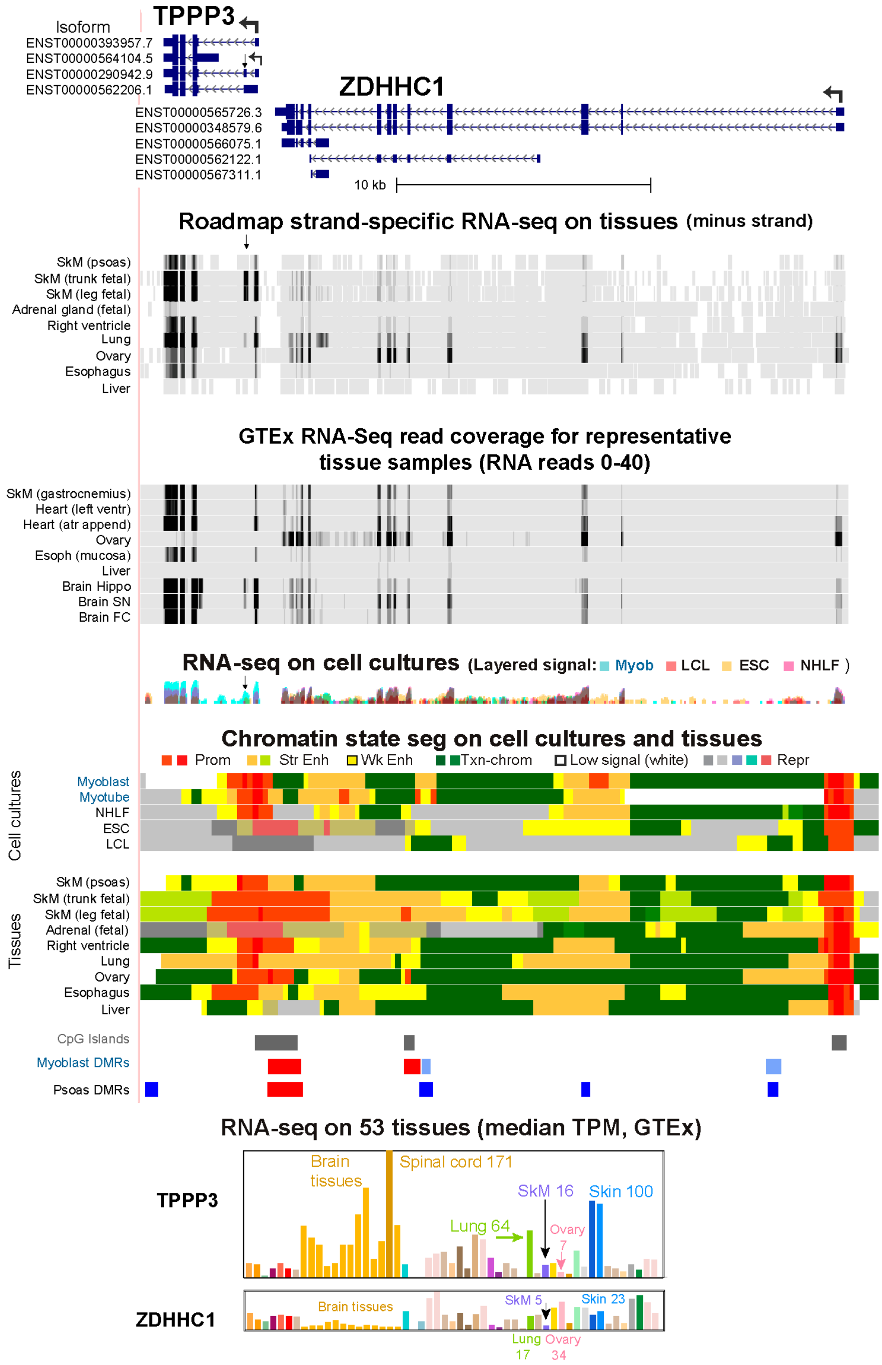

Figure S12. ***TPPP3* and its upstream neighbor *ZDHHC1*.** The region shown, chr16:67,388,887-67,417,847 (hg38), is the same as that of Figure 6B, but this figure includes tissue RNA-seq profiles (Roadmap Epigenomics Project) and additional gene isoforms. Small vertical arrows at the gene structures, Roadmap RNA-seq, and cell culture RNA-seq tracks, the position of the alternative exon 2, which is missing in postnatal psoas muscle but present in fetal SkM and myoblasts. Light blue Myob-hypom DMR bars, the absolute value of methylation difference was 0.25 – 0.35 unlike >0.35 for all the other DMRs shown.
